# Keratinocytes costimulate naive human T cells via CD2: a potential target to prevent the development of proinflammatory Th1 cells in the skin

**DOI:** 10.1038/s41423-019-0261-x

**Published:** 2019-07-19

**Authors:** Christian Orlik, Daniel Deibel, Johanna Küblbeck, Emre Balta, Sabina Ganskih, Jüri Habicht, Beate Niesler, Jutta Schröder-Braunstein, Knut Schäkel, Guido Wabnitz, Yvonne Samstag

**Affiliations:** 1 Institute of Immunology, Section Molecular Immunology, Heidelberg University, Im Neuenheimer Feld 305, 69120 Heidelberg, Germany; 2 Institute of Human Genetics, Department of Human Molecular Genetics, and nCounter Core Facility, Heidelberg University, Im Neuenheimer Feld 366, 69120 Heidelberg, Germany; 3 Department of Dermatology, Heidelberg University, Im Neuenheimer Feld 440, 69120 Heidelberg, Germany

**Keywords:** keratinocytes, inflammatory skin diseases, psoriasis, human T cells, costimulation, CD2, LFA-1, nonprofessional antigen-presenting cells, Th1 cells, Th17 cells, Autoimmunity, Lymphocyte activation

## Abstract

The interplay between keratinocytes and immune cells, especially T cells, plays an important role in the pathogenesis of chronic inflammatory skin diseases. During psoriasis, keratinocytes attract T cells by releasing chemokines, while skin-infiltrating self-reactive T cells secrete proinflammatory cytokines, e.g., IFNγ and IL-17A, that cause epidermal hyperplasia. Similarly, in chronic graft-versus-host disease, allogenic IFNγ-producing Th1/Tc1 and IL-17-producing Th17/Tc17 cells are recruited by keratinocyte-derived chemokines and accumulate in the skin. However, whether keratinocytes act as nonprofessional antigen-presenting cells to directly activate naive human T cells in the epidermis remains unknown. Here, we demonstrate that under proinflammatory conditions, primary human keratinocytes indeed activate naive human T cells. This activation required cell contact and costimulatory signaling via CD58/CD2 and CD54/LFA-1. Naive T cells costimulated by keratinocytes selectively differentiated into Th1 and Th17 cells. In particular, keratinocyte-initiated Th1 differentiation was dependent on costimulation through CD58/CD2. The latter molecule initiated STAT1 signaling and IFNγ production in T cells. Costimulation of T cells by keratinocytes resulting in Th1 and Th17 differentiation represents a new explanation for the local enrichment of Th1 and Th17 cells in the skin of patients with a chronic inflammatory skin disease. Consequently, local interference with T cell–keratinocyte interactions may represent a novel strategy for the treatment of Th1 and Th17 cell-driven skin diseases.

## Introduction

The crosstalk between keratinocytes (KCs) and T cells plays a pivotal role during the development of chronic inflammatory skin diseases.^1^ In psoriasis, an autoimmune skin disease that affects ~2% of the world population, T cell infiltration into the skin and epidermal hyperplasia are key histological features.^2,3^ Thereby, self-reactive T cells, which are activated in skin-draining lymph nodes by antigen-presenting cells (APCs), infiltrate the skin and secrete proinflammatory cytokines, e.g., interleukin (IL)-17 and IFNγ.^4,5^ In particular, IL-17 family cytokines induce KC proliferation and inhibit KC differentiation.^6,7^ Specifically, in the early phase of the pathogenic cascade, IL-17 together with IL-22 and IFNγ drives a feed-forward loop. In response to these cytokines, KCs secrete chemokines (CCL19, CCL20, CXCL1–3, CXCL9, and CXCL10), leading to the recruitment of neutrophils, IFNγ- or IL-17-producing T cells, and mature plasmacytoid or myeloid dendritic cells (DCs).^8–11^ In turn, these highly inflammatory skin-infiltrating DCs cause the activation of autoreactive T cells during the amplification phase, thereby boosting inflammation.^12,13^

In addition, in chronic graft-versus-host disease (GVHD), a major complication of allogenic stem cell transplantation, the KC-mediated secretion of chemokines (CXCL9 and CXCL10) leads to the recruitment of alloreactive T cells into the skin.^14^ These allogenic T cells predominantly belong to the IFNγ-producing Th1/Tc1 and IL-17-producing Th17/Tc17 subpopulations and cause cutaneous manifestations, e.g., follicular erythema.^15–17^

Although the pivotal role of KCs in non-contact-mediated communication during chronic skin inflammation is quite well understood, the direct interaction between KCs and T cells remains elusive. In particular, the potential of KCs to act as nonprofessional APCs, enabling them to costimulate T cells directly in the skin, is still debated.

T cells require two distinct signals for activation and clonal expansion. The first signal is transmitted by the antigen-specific T cell receptor (TCR) on T cells, following recognition of antigenic peptides loaded on MHC class I or class II molecules expressed by APCs. The first signal secures the antigen specificity of the immune reaction. The second signal is transmitted through costimulatory receptors, dictating the progression to T cell activation. Between professional APCs (pAPCs) and T cells, the costimulatory signal is largely delivered by the interaction of CD80 or CD86 on the pAPC and CD28 on the T cell.^18^ In addition, other receptor pairs, e.g., CD58/CD2 and CD54/LFA-1, can transmit costimulatory signals between professional APCs and T cells.^19,20^ In the absence of costimulation, antigen recognition leads to anergy or even apoptosis in T cells.^21,22^ Since unstimulated KCs do not express CD80/CD86 or MHC class II molecules on their cell surface, it has been widely believed that KCs are unable to costimulate naive T cells.^23^ However, KCs upregulate the expression of MHC class II molecules in response to IFNγ, so that they are able to present antigens not only to CD8^+^ cytotoxic T cells but also to CD4^+^ helper T cells.^24–26^ Some studies have provided evidence that the exposure of KCs to autoantigens and viral or bacterial products, at least under proinflammatory conditions, initiates the activation of memory T cells through a direct interaction.^26,27^ These studies, however, used either preactivated T cells or peripheral blood mononuclear cells (PBMCs) containing pAPCs, e.g., monocytes or DCs. In addition, none of these studies addressed the question of whether KCs can modulate further T cell differentiation.

To clarify whether in the absence of pAPCs, KCs can provide costimulatory signals to naive T cells, we established a coculture system with primary human KCs and unstimulated primary human T cells. Our experiments demonstrated that IFNγ-pretreated primary human KCs were indeed able to activate even naive T cells by providing costimulatory signals via CD58/CD2 and CD54/LFA-1 interactions. Notably, these T cells selectively differentiated into Th1 and Th17 cells, which are known to be involved in the pathogenesis of psoriasis, as well as the cutaneous manifestation of chronic GVHD.

## Results

### IFNγ-pretreated keratinocytes activate human peripheral blood T cells in the absence of DCs

**Fig. 1:**
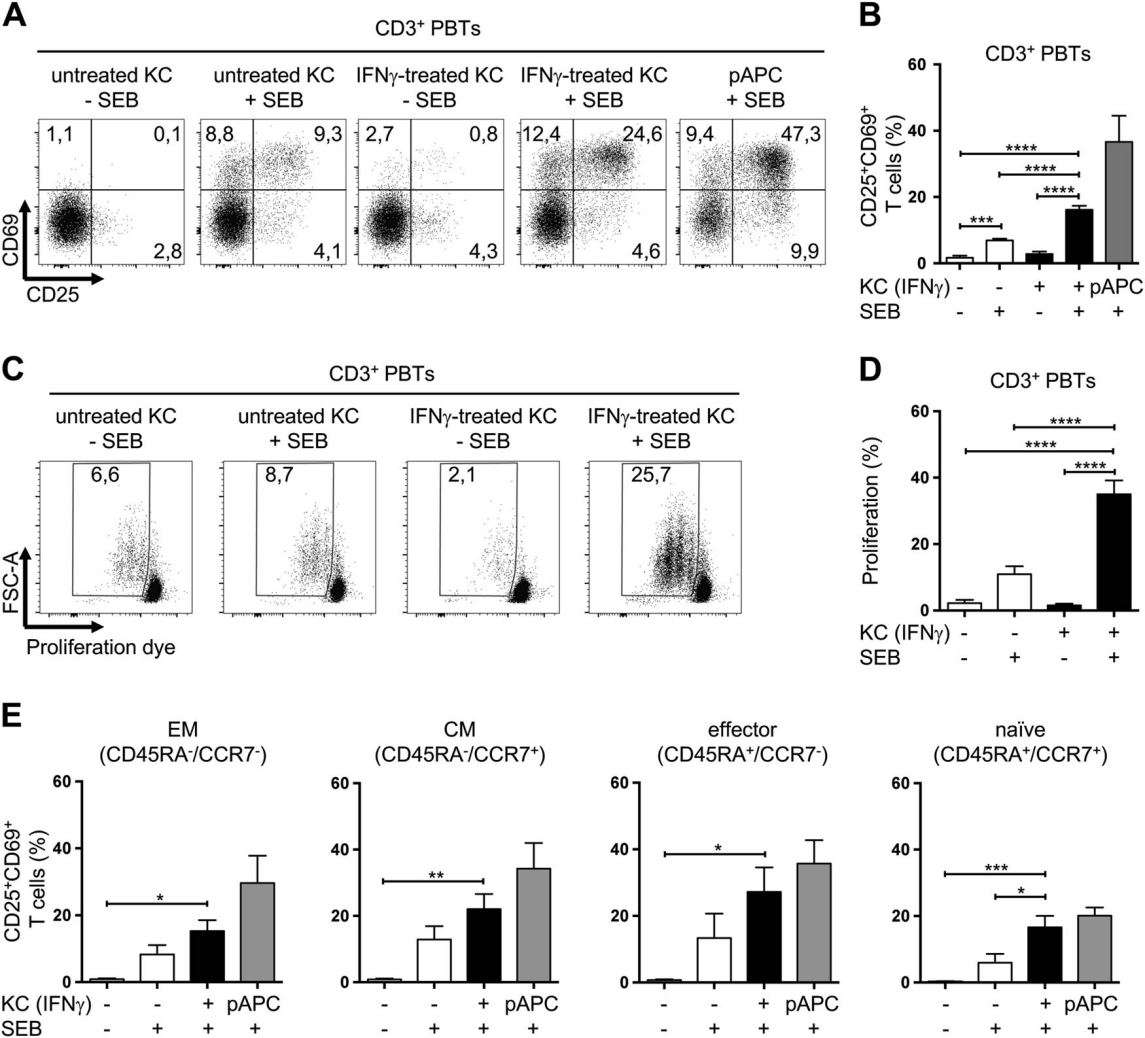
IFNγ-pretreated keratinocytes (KCs) stimulate peripheral blood T cells (PBTs). CD3^+^ PBTs were cocultured for the indicated time points with untreated KCs (white bars) or IFNγ-pretreated KCs (black bars) loaded with SEB (+) or not (−) and then analyzed by flow cytometry. Professional antigen-presenting cells (pAPCs) loaded with SEB served as a positive control (gray bars). Representative dot plots (**a**) and statistical evaluation (**b**) of CD25 and CD69 expression after 24 h of coculture (*n* = 10 individual T cell donors). Representative dot plots (**c**) and statistical evaluation (**d**) of the proliferation of CD3^+^ PBTs cocultured for 72 h (*n* = 6 individual T cell donors). Proliferation was assessed by a CFDA dilution assay. **e** CD25 and CD69 expression of T cell subpopulations after 24 h of coculture (*n* = 5 individual T cell donors). CD3^+^ PBTs were categorized into effector memory (EM, CD45RA^−^CCR7^−^), central memory (CM, CD45RA^−^CCR7^+^), effector (effector, CD45RA^+^CCR7^−^), and naive (naive, CD45RA^+^CCR7^+^) PBTs. Data are represented as the mean ± SEM. ****p < 0.0001; ***p < 0.001; **p < 0.01; and *p < 0.05. See also Fig. S1

To address the question of whether primary human KCs are able to activate unstimulated human naive T cells, we first established an in vitro coculture system with primary human KCs and untransformed human peripheral blood T cells (PBTs). To mimic proinflammatory conditions and to induce the expression of MHC class II molecules, KCs were pretreated with type II IFN (IFNγ) for 24 h (Fig. S1A). Of note, other cytokines found in lesions in inflammatory skin diseases did not induce HLA-DR expression on primary KCs (Fig. S1B). Then, the IFNγ was removed, and the KCs were loaded with a superantigen of *Staphylococcus aureus* (staphylococcal enterotoxin B (SEB)) that enables polyclonal cross-linking of HLA-DR with the TCR, mimicking antigen recognition by the TCR.^28–30^ Subsequently, PBTs were added to either untreated or IFNγ-pretreated SEB-loaded KCs. Stimulation with SEB-loaded Raji cells, which were used as pAPCs, served as a positive control. T cell activation was then assessed by analyzing the expression of the activation markers CD25 (IL-2Rα) and CD69 on the cell surface using flow cytometry (Fig. 1a, b). SEB loading of KCs without further pretreatment led to minor inductions of both activation markers, whereas IFNγ pretreatment strongly enhanced the capacity of SEB-loaded KCs to induce T cell activation. In line with the enhanced expression of CD25, IFNγ-pretreated SEB-loaded KCs significantly enhanced the induction of T cell proliferation compared with untreated SEB-loaded KCs (Fig. 1c, d). This KC-mediated T cell activation was dependent on the SEB-mediated cross-linking between HLA-DR and the TCR, since prior siRNA-mediated knockdown of HLA-DR expression (Fig. S1C) significantly diminished T cell activation (Fig. S1D). Accordingly, neither untreated nor IFNγ-pretreated KCs were able to stimulate PBTs in the absence of SEB (Fig. 1a–d, S1E). A more detailed analysis revealed that CD4^+^ and CD8^+^ T cells were equally activated by IFNγ-pretreated KCs loaded with SEB (Fig. S1E).

**Fig. 2:**
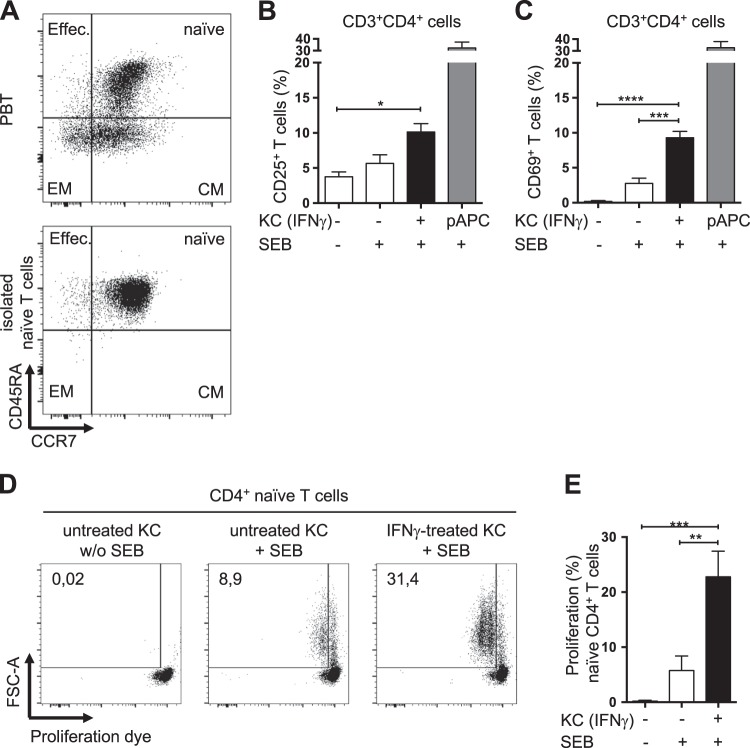
IFNγ-pretreated keratinocytes activate naive T cells. **a** Representative dot plots of the surface expression of CD45RA and CCR7 on CD3^+^ PBTs (upper panel) and isolated naive CD4^+^ T cells (lower panel), as assessed by flow cytometry. **b**–**e** Naive CD4^+^ T cells were cultured for the indicated time periods with untreated KCs (white bars) or IFNγ-pretreated KCs (black bars) loaded with SEB (+) or not (−), or with professional antigen-presenting cells (pAPCs) loaded with SEB as a positive control (gray bars) and then analyzed by flow cytometry. Statistical evaluation of CD25 (**b**) and CD69 (**c**) expression after 24 h of coculture (*n* = 6 individual T cell donors). Representative dot plots (**d**) and statistical evaluation (**e**) of the proliferation of naive CD4^+^ T cells cocultured for 72 h (*n* = 6 individual T cell donors). Proliferation was assessed by a CFDA dilution assay. Data are represented as the mean ± SEM. ****p < 0.0001; ***p < 0.001; **p < 0.01; *p < 0.05. See also Fig. S2

Next, we investigated whether naive T cells within the PBT population were also activated by IFNγ-pretreated KCs. According to their expression of CD45RA and CCR7, PBTs were categorized as effector, memory, or naive T cells (Fig. 2a, upper dot plot).^31–33^ Indeed, following stimulation with IFNγ-pretreated KCs, CD25^+^CD69^+^-activated T cells were found not only in the memory (Fig. 1e; EM/CM, CD45RA^−^) and effector T cell populations (Fig. 1e; effector, CD45RA^+^CCR7^–^) but also in the naive T cell population (Fig. 1e; naive, CD45RA^+^CCR7^+^). Notably, no contaminating pAPCs were found within the isolated KC and T cell populations (Fig. S1F, G). However, these experimental settings do not exclude the potential for different T cell subsets within the PBT population, especially the memory T cell subset, to influence KC-dependent activation of naive T cells. Consequently, further experiments with isolated naive T cells were performed.

### IFNγ-pretreated keratinocytes activate naive human T cells in the absence of DCs

Isolated (negatively selected) naive CD4^+^ T cells (Fig. 2a) were cocultured with untreated or IFNγ-pretreated SEB-loaded KCs as described above. Similar to PBTs, the isolated naive T cells showed a significant induction of CD25 and CD69 expression after coculture with IFNγ-pretreated KCs, and this induction was again higher than that observed after coculture, with either unloaded or SEB-loaded untreated KCs (Fig. 2b, c). Notably, this KC-dependent T cell activation was highly dependent on the cross-linking of HLA-DR and the TCR, since prior HLA-DR knockdown resulted in drastically decreased T cell activation (Fig. S2). Consistent with the surface expression pattern of CD25, IFNγ-pretreated SEB-loaded KCs were able to initiate strong proliferation in naive T cells (Fig. 2d, e). These data suggest that KCs can indeed function as nonprofessional APCs.

### CD54/LFA-1 and CD58/CD2 interactions are required for keratinocyte-dependent T cell stimulation

**Fig. 3:**
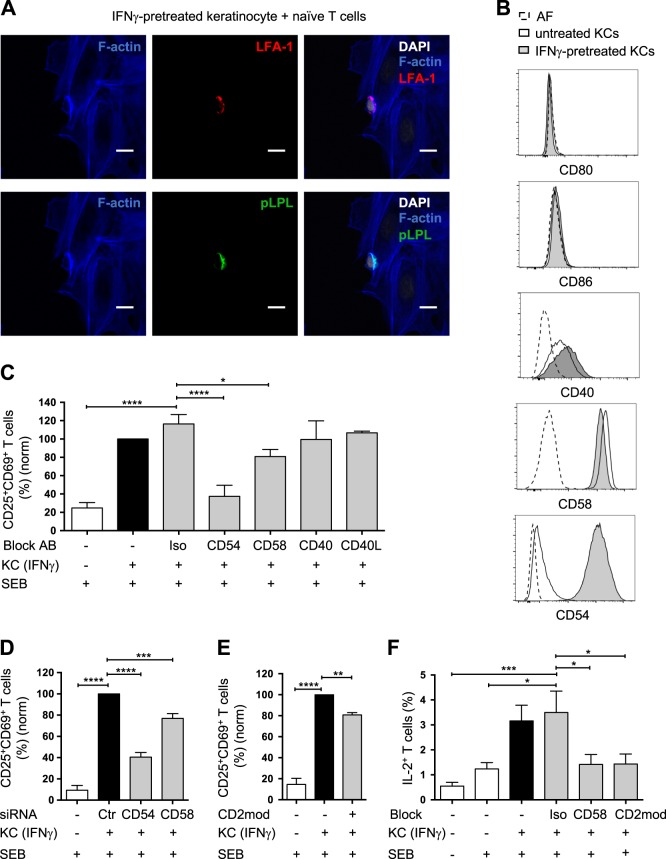
CD54/LFA-1 and CD58/CD2 interactions are needed for keratinocyte-dependent T cell activation. **a** Representative confocal microscopy image of naive CD4^+^ T cells cultured for 30 min with IFNγ-pretreated SEB-loaded KCs. **Upper panel:** immunofluorescence staining for F-actin (blue) and LFA-1 (red) with DAPI staining (white). **Lower panel:** immunofluorescence staining for F-actin (blue) and phosphorylated L-plastin (pLPL, green) with DAPI staining (white). Scale bar = 10 µm. **b** Representative histograms of the surface expression of costimulatory receptors on untreated KCs (black line, white background) and KCs pretreated with IFNγ for 24 h (black line, gray background). Untreated KCs stained with an isotype control antibody (AF): dashed line, white background. **c**–**f** Naive CD4^+^ T cells were cultured for 24 h with untreated KCs (white bars) or IFNγ-pretreated KCs (black and gray bars) loaded with SEB (+) and then analyzed by flow cytometry. The effect of isotype control antibodies (Iso), blocking antibodies against costimulatory receptors (CD54, CD58, CD40, and CD40L) (**c**), siRNA treatment of KCs (control (Ctr), CD54-specific, or CD58-specific siRNAs) (**d**), or CD2 downmodulation (CD2mod) in T cells (**e**) on CD25 and CD69 expression after 24 h of coculture (*n* = 6 individual T cell donors). The data were normalized to the percentage of CD25^+^CD69^+^ naive CD4^+^ T cells cultured with IFNγ-pretreated KCs loaded with SEB (black bar). **f** Effect of blocking antibodies against CD58 or CD2 downmodulation (CD2mod) in T cells on intracellular IL-2 expression after 24 h of coculture (*n* = 6 individual T cell donors). Data are represented as the mean ± SEM. ****p < 0.0001; ***p < 0.001; **p < 0.01; *p < 0.05. See also Fig. S3

Costimulation of T cells by pAPCs induces the formation of a mature immunological synapse (IS) at the contact zone between the cells.^34^ The IS consists of defined and in-part stable structures that are involved in the transmission of signals through TCR/MHC interactions and costimulatory receptors. We investigated the contact zone between naive human T cells and IFNγ-pretreated SEB-loaded primary KCs. As described for the contact zone between pAPCs and naive T cells, filamentous actin (F-actin) and LFA-1 accumulated in the contact zone between the IFNγ-pretreated KCs and naive T cells (Fig. 3a, upper panel), indicating the formation of a mature IS. In addition, L-plastin (LPL), an actin-bundling protein that is associated with the stability of the IS, appeared in its phosphorylated form at the interaction site between the IFNγ-pretreated KCs and naive T cells (Fig. 3a, lower panel). Since LPL phosphorylation at the IS requires costimulation,^35^ the accumulation of phosphorylated LPL (pLPL) in the contact zone between the IFNγ-pretreated KCs and naive T cells indicated the ability of the KCs to deliver costimulatory signals.

To identify the participating costimulatory receptors, costimulatory receptor expression on untreated and IFNγ-pretreated KCs was analyzed using flow cytometry. In line with previous reports, our study showed that neither untreated nor IFNγ-pretreated KCs expressed CD80 or CD86 (Fig. 3b).^26^ However, we found that KCs expressed high levels of CD40 and CD58 on the cell surface, regardless of whether they were pretreated with IFNγ (Fig. 3b). In addition, as described earlier, IFNγ-pretreated KCs expressed high levels of CD54, whereas untreated KCs showed no CD54 expression (Fig. 3b). Additional costimulatory receptors were not detected on the surface of untreated or IFNγ-pretreated KCs (Table S1).

To further determine which of the detected costimulatory receptors were required to deliver costimulatory signals to naive T cells, antibodies blocking the interaction between these receptors and their ligands were used. We observed strongly decreased activation of naive T cells cocultured with IFNγ-pretreated KCs upon blockade of CD54 (Fig. 3c), which is in line with previous results from experiments performed with IFNγ-pretreated KCs and memory T cells.^26^ This decrease was probably due to a loss of T cell adherence to the IFNγ-pretreated KCs (Fig. S3A, B). In contrast, blocking CD40 and its ligand CD40L did not influence KC-mediated T cell activation (Fig. 3c). Importantly, the blockade of CD58 on IFNγ-pretreated SEB-loaded KCs caused significantly reduced activation of naive T cells (Fig. 3c), without disturbing T cell adherence to the KCs (Fig. S3A, B). These findings were independently confirmed by using an siRNA approach. Knocking down CD54 or CD58 expression on KCs using appropriate siRNAs (Fig. S3C) resulted in reductions in the KC-mediated activation of naive T cells similar to those observed in the antibody-mediated blockade experiments (Fig. 3d).

Given the observed accumulation of LFA-1 in the contact zone between naive T cells and IFNγ-pretreated KCs, the CD54/LFA-1 interaction is probably involved in the initiation and stabilization of the contact zone between T cells and KCs. However, an analysis of LFA-1-dependent costimulatory signals in our coculture system was not possible, since upon CD54 blockade (antibody or siRNA mediated), contact between T cells and IFNγ-pretreated KCs was lost (Fig. S3A, B).

To further investigate whether KCs transmit costimulatory signals through the interaction of CD58 with its ligand CD2 on T cells, the surface expression of CD2 on naive T cells was downregulated by using a modulating CD2-specific IgM antibody (CD2mod) (Fig. S3D, E). Indeed, naive T cells with reduced CD2 surface expression showed significantly reduced KC-mediated activation, as measured by the expression of CD25 and CD69 (Fig. 3e).

To finally clarify whether the interaction of CD58 and CD2 plays a key role in the costimulation of naive T cells by IFNγ-pretreated KCs, we analyzed the expression of IL-2, a costimulation-dependent cytokine (Fig. 3f).^36,37^ While the coculture of naive T cells with IFNγ-pretreated KCs clearly induced the production of IL-2 by the T cells, blocking CD58 or CD2 abolished KC-initiated IL-2 production (Fig. 3f). These results demonstrate the importance of CD58 and CD2 in costimulatory signaling during the KC-mediated activation of naive T cells.

### IFNγ-pretreated keratinocytes selectively initiate the differentiation of naive T cells into Th1 and Th17 cells

**Fig. 4:**
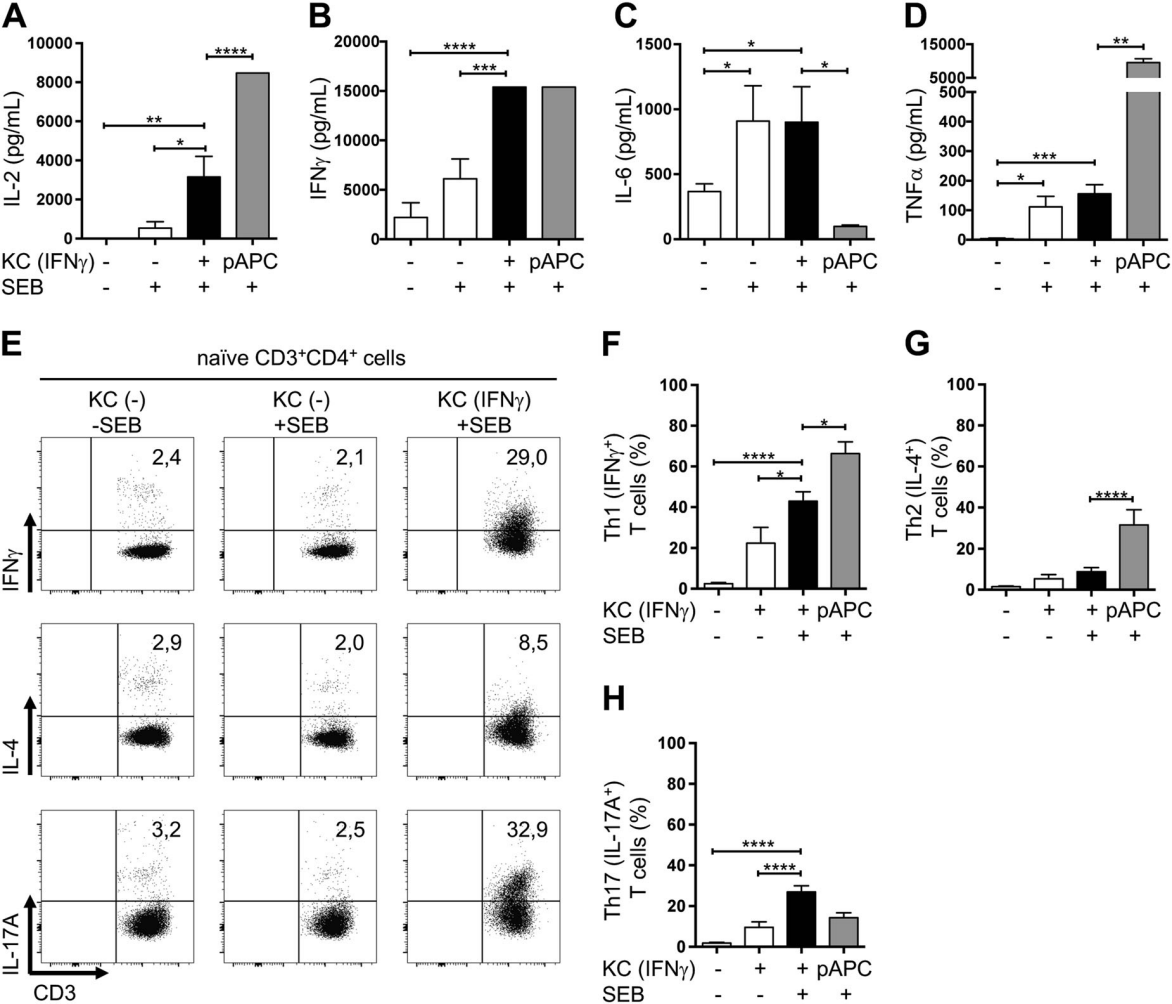
IFNγ-pretreated keratinocytes initiate T cell-specific cytokine production, triggering Th1 and Th17 differentiation. Naive CD4^+^ T cells were cultured for the indicated time periods with untreated KCs (white bars) or IFNγ-pretreated KCs (black bars) loaded with SEB (+) or not (−) and then analyzed by flow cytometry. Professional antigen-presenting cells (pAPCs) loaded with SEB served as a positive control (gray bars). Amounts of secreted IL-2 (**a**), IFNγ (**b**), IL-6 (**c**), and TNFα (**d**) after 24 h of coculture (*n* = 6 individual T cell donors). Secreted cytokine amounts were analyzed by a cytokine bead array. Representative dot plots (**e**) and statistical evaluation of the differentiation into Th1 (IFNγ^+^) cells (**f**), Th2 (IL-4^+^) cells (**g**), and Th17 (IL-17A^+^) cells (**h**) after 6 days of coculture (*n* = 5 individual T cell donors). Intracellular cytokine expression was analyzed after PMA/ionomycin treatment. Data are represented as the mean ± SEM. ****p < 0.0001; ***p < 0.001; **p < 0.01; *p < 0.05. See also Fig. S4

We next investigated whether KC–T cell interactions produce a particular cytokine milieu that determines the fate and subsequent differentiation of naive T cells. To address this question, the secretion of cytokines involved in the differentiation processes of distinct T cell subsets was analyzed in the supernatants of a 24 h coculture of KCs and naive T cells. In addition to IL-2, the proinflammatory cytokine IFNγ was strongly secreted in the coculture of naive T cells and IFNγ-pretreated KCs (Fig. 4a, b). Of note, the IFNγ used for KC pretreatment was removed before initiating the T cell–KC coculture (Fig. S4B). Along with IFNγ, which is involved in the Th1 differentiation process, IL-6, which is involved in Th17 differentiation, was highly secreted into the supernatant (Fig. 4c). Interestingly, except for IL-10, which was present in very low amounts, cytokines linked to the differentiation of other Th subsets, e.g., IL-4 (Th2 cells), IL-9 (Th9 cells), and IL-22 (Th22 cells), and effector cytokines (IL-17A and IL-17F) were not detectable in the coculture between naive T cells and either untreated or IFNγ-pretreated KCs (Fig. S4A). While the amounts of IL-2 and IFNγ were similar after stimulation of naive T cells, with either IFNγ-pretreated KCs or pAPCs, only a minor amount of TNFα secretion was observed in the supernatants of T cells cocultured with IFNγ-pretreated KCs compared with those of T cells cocultured with pAPCs (Fig. 4d). Conversely, IL-6 was detected in the supernatants derived from KC–T cell cocultures; however, it was not found in the supernatants of T cells cocultured with pAPCs (Fig. 4c). These findings indicate that the functional outcome differs, depending on whether naive T cells are costimulated by KCs or pAPCs.

The particular nature of the observed cytokine milieu suggests that IFNγ-pretreated KCs may specifically trigger the polarization of naive T cells toward Th1 and Th17 differentiation. We tested this hypothesis by coculturing naive T cells with untreated or IFNγ-pretreated SEB-loaded KCs for 6 days. Thereafter, T cells were restimulated with PMA/ionomycin, and the production of cytokines and expression of transcription factors characteristic of Th1, Th2, or Th17 cells were determined by flow cytometry. We found drastically increased production of IFNγ (Th1-associated) and IL-17A (Th17-associated) in the T cells cocultured with IFNγ-pretreated KCs compared with the T cells cocultured with untreated KCs (Fig. 4e (upper and lower panels, respectively), f, and h). In contrast, the Th2-associated cytokine IL-4 was hardly detectable in the T cells cocultured for 6 days with untreated or IFNγ-pretreated KCs (Fig. 4f (middle panel) and g).

To independently validate whether KCs initiate selective differentiation into Th1 and Th17 cells, the expression of the transcription factors T-bet, GATA-3, and RORγt, which are functionally linked to Th1, Th2, and Th17 cell differentiation, respectively, was determined. In line with the observed cytokine pattern, the expression of T-bet (Fig. S4C, D) and RORγt (Fig. S4C, F), but not that of GATA-3 (Fig. S4C, E), was highly enhanced in the T cells cocultured for 6 days with IFNγ-pretreated KCs compared with the T cells cocultured with untreated KCs.

Interestingly, compared with the coculture with IFNγ-pretreated KCs, the 6-day coculture of pAPCs and naive T cells initiated higher frequencies of IFNγ (Th1)- and IL-4 (Th2)-producing T cells, but a lower frequency of IL-17 (Th17)-producing T cells (Fig. 4f–h). Together, these results indicated that the interaction of primary IFNγ-pretreated KCs with naive T cells generated a cytokine milieu that preferentially triggered Th1 and Th17 differentiation, whereas coculture with pAPCs preferentially triggered differentiation into Th1 and Th2 cells.

### Keratinocyte-initiated Th1 differentiation is dependent on costimulation via CD58/CD2

**Fig. 5:**
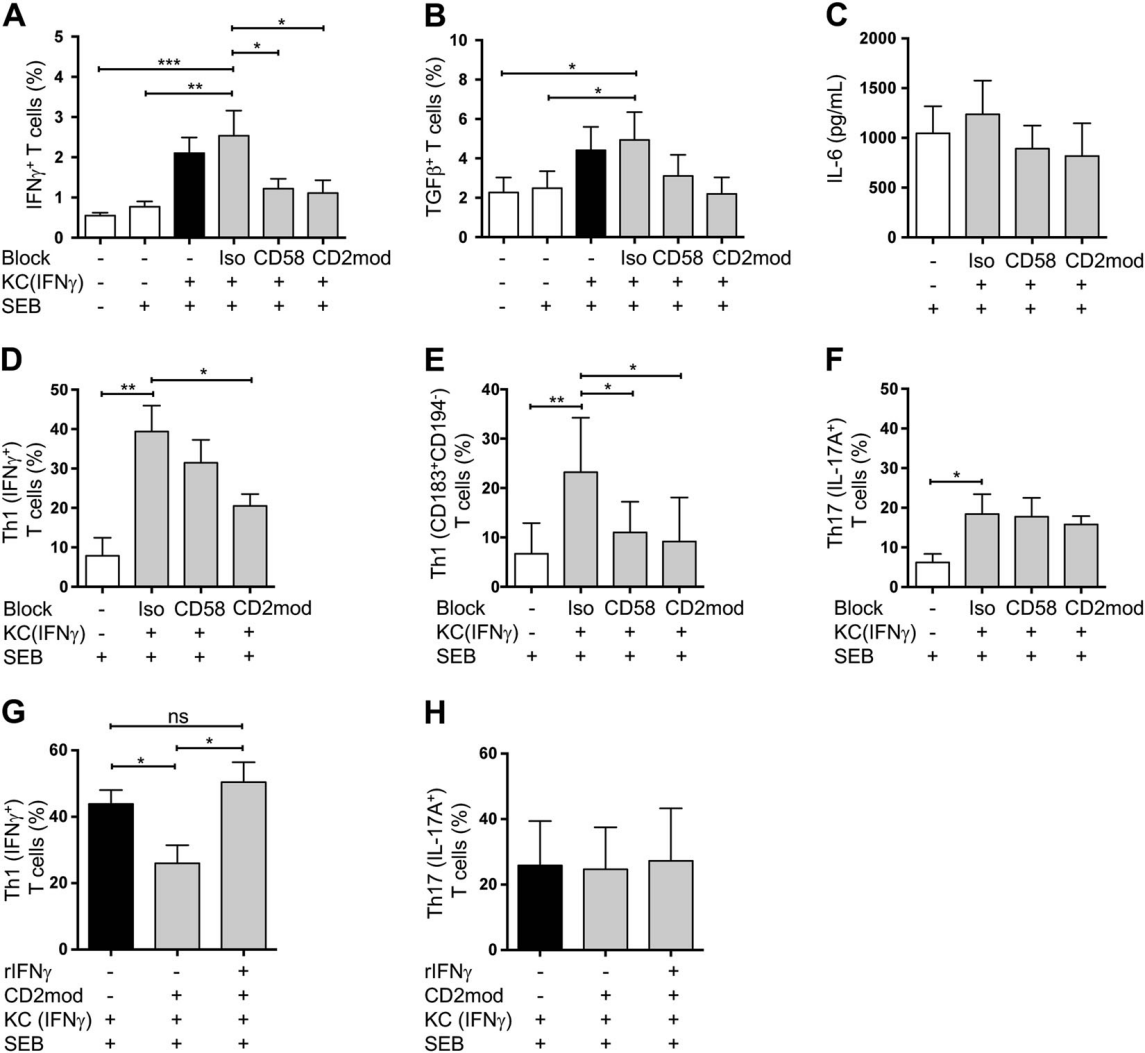
Keratinocyte-initiated Th1 differentiation is dependent on costimulation through CD58/CD2. Naive CD4^+^ T cells were cultured for the indicated time periods with untreated KCs (white bars) or IFNγ-pretreated KCs (black and gray bars) loaded with SEB (+) or not (−). Cytokine expression in the T cells was analyzed by flow cytometry, or cytokine secretion into the supernatants was determined by a cytokine bead array. Effects of isotype control antibodies (Iso), blocking antibodies against CD58, or CD2 downmodulation (CD2mod) in T cells on intracellular IFNγ expression (**a**), TGFβ expression (**b**), and IL-6 secretion into the supernatant (**c**) after 24 h of coculture (*n* ≥ 6 individual T cell donors). Effects of isotype control antibodies (Iso), blocking antibodies against CD58, or CD2 downmodulation (CD2mod) in T cells on the differentiation of Th1 (IFNγ^+^) cells (**d**), Th1 (CD183^+^CD194^−^) cells (**e**), and Th17 (IL-17A^+^) (**f**) after 6 days of coculture. Intracellular cytokine expression and CD183 and CD194 surface expression were analyzed after PMA/ionomycin treatment (*n* ≥ 6 individual T cell donors). Effects of exogenously added recombinant IFNγ (rIFNγ) on Th1 (IFNγ^+^) (**g**) and Th17 (IL-17A^+^) (**h**) differentiation after CD2 downmodulation (CD2mod) in a 6-day coculture (*n* = 4 individual T cell donors). Intracellular cytokine expression was analyzed after PMA/ionomycin treatment. Data are represented as the mean ± SEM. ***p < 0.001; **p < 0.01; *p < 0.05; and ns not significant. See also Fig. S5

As demonstrated above, IFNγ-pretreated primary human KCs, which naturally lack the expression of CD80/CD86, were able to costimulate naive human T cells via the interaction of CD58 (on KCs) with CD2 (on T cells), resulting in IL-2 production by T cells and T cell proliferation (Figs. 3f, 2e). We next tested whether costimulation by CD58/CD2 differentially regulates cytokines related to the above-mentioned KC-induced T cell differentiation. Preventing the CD58/CD2 interaction prior to coculturing T cells with IFNγ-pretreated KCs, either by preincubation with a blocking anti-CD58 antibody (CD58) or by antibody-mediated downmodulation of CD2 (CD2mod), diminished the early induction of IFNγ production in T cells (within 24 h) compared with treatment with isotype control antibodies (Iso) (Fig. 5a). Since the specific cytokine milieu ultimately dictates T cell differentiation, this finding indicated that the CD58/CD2 interaction may be important in Th1 differentiation. In contrast, transforming growth factor β (TGFβ), a cytokine crucially involved in Th17 differentiation,^38^ was equally produced in naive T cells cultured with IFNγ-pretreated KCs, regardless of whether CD58/CD2 signaling was blocked (Fig. 5b). IL-6, another cytokine involved in Th17 differentiation, was secreted by untreated and IFNγ-pretreated KCs rather than by T cells (Fig. S5A, B), and was also not influenced by blocking the CD58/CD2 interaction (Fig. 5c).

Next, we analyzed T cell differentiation following coculture with IFNγ-pretreated KCs for 6 days. Upon blockade of CD58 or CD2, fewer Th1 cells were found in the blocking antibody-treated cocultures than in the cocultures of cells pretreated with isotype control antibodies (Fig. 5d). Consistent with these results, the expression of surface markers associated with Th1 cells (CD183^+^CD194^−^, reviewed in ref. ^39^) was induced upon coculture with IFNγ-pretreated KCs, but significantly diminished after blockade of CD58 and CD2, compared with coculture in the presence of an isotype control antibody (Fig. 5e). In contrast, the amount of Th17 cells was not affected by blocking CD58/CD2 signaling (Fig. 5f). Together, these data reveal an important role for costimulation through CD58/CD2 in KC-initiated Th1 differentiation, while this costimulation was not involved in Th17 polarization.

One plausible explanation for the diminished Th1 differentiation upon CD58/CD2 blockade could be diminished IFNγ production within the first 24 h (compare Fig. 5a). To address this point, recombinant IFNγ (rIFNγ) was exogenously added to a coculture of IFNγ-pretreated KCs and CD2-downmodulated naive T cells, and T cell differentiation was assessed after 6 days. Indeed, supplementation with rIFNγ rescued the diminished Th1 differentiation caused by blocking CD58/CD2 costimulation (Fig. 5g). As expected, this treatment did not influence Th17 differentiation (Fig. 5h). Together, these results demonstrate a clear relationship between costimulatory signaling via CD58/CD2 during the interaction of primary KCs with naive T cells and the generation of a cytokine milieu favoring Th1 differentiation.

### IFNγ-pretreated keratinocytes induce STAT1 expression and phosphorylation in naive T cells through CD58/CD2 costimulation

**Fig. 6:**
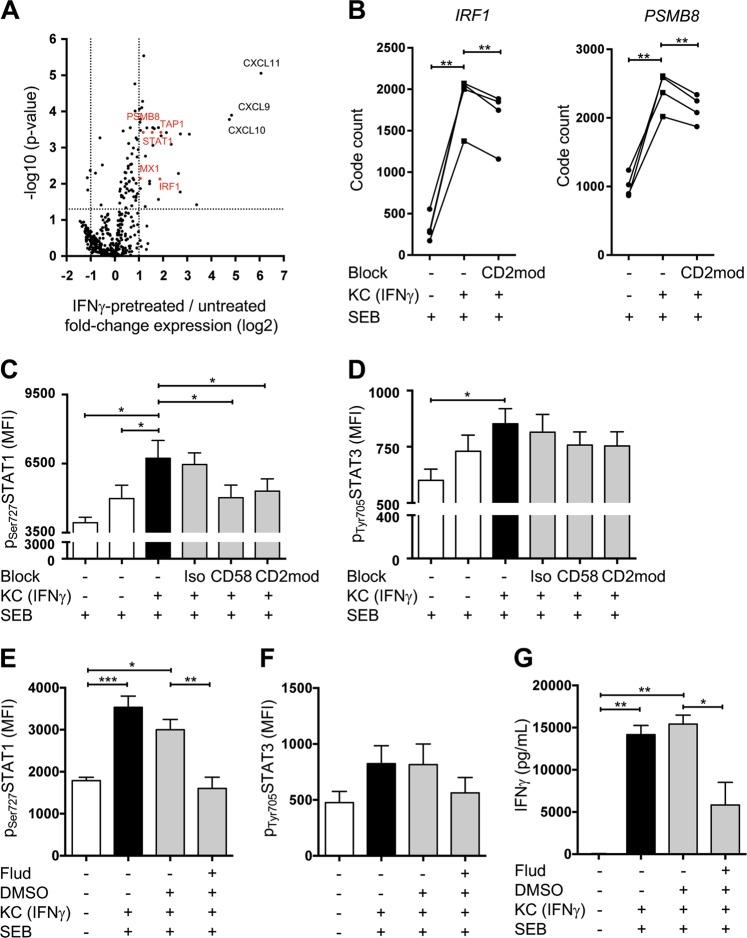
IFNγ-pretreated keratinocytes initiate STAT1 phosphorylation in naive T cells through CD58/CD2 costimulation. Naive CD4^+^ T cells were cultured for the indicated time periods with untreated KCs (white bars) or IFNγ-pretreated KCs (black and gray bars) loaded with SEB and then analyzed by nCounter GEx (mRNA expression) and flow cytometry**. a** Volcano plot of the fold change in the mRNA code counts (log-2 transformed) plotted against its significance (–log_10_-transformed *p* value). Fold changes were calculated from the mRNA code counts isolated from naive CD4^+^ T cells cultured with IFNγ-pretreated KCs compared with those from naive CD4^+^ T cells cultured with untreated KCs loaded with SEB for 4 h (red dots indicate STAT1-regulated genes). **b** Effects of CD2 downmodulation (CD2mod) on the mRNA code counts of *IRF1* and *PSMB8* in a 4-h coculture (*n* = 4 individual T cell donors; each dot represents a single data point derived from individual T cell donors). mRNA expression was analyzed by Nanostring (Seattle, Washington; nCounter human Immunology Panel). Effects of isotype control antibodies (Iso), blocking antibodies against CD58, or CD2 downmodulation (CD2mod) in T cell-specific STAT1 (**c**) and STAT3 (**d**) phosphorylation (*n* = 5 individual T cell donors) after 24 h of coculture. Effect of fludarabine (Flud) treatment on T  cell-specific STAT1 (**e**) and STAT3 (**f**) phosphorylation or IFNγ secretion into the supernatant (**g**) (*n* = 4 individual T cell donors) after 24 h of coculture. Data are represented as the mean ± SEM. ***p < 0.001; **p < 0.01; *p < 0.05. See also Fig. S6

Next, we aimed to understand the molecular basis of the correlations between T cell-specific IFNγ production and subsequent Th1 polarization, and CD58/CD2-mediated costimulation by KCs. To address this issue, total RNA was purified from naive T cells that were cultured with either untreated or IFNγ-pretreated KCs for 4 h, and the expression of ~600 genes associated with immunological pathways was assessed by the nCounter platform of Nanostring. Forty-six genes had strongly upregulated expression in the naive T cells cocultured with IFNγ-pretreated KCs compared with the naive T cells cocultured with untreated KCs (Fig. S6A). Differential expression analysis of transcripts derived from five individual donors and subsequent ingenuity pathway analysis predicted that the upregulated genes were associated with pathways linked to Th1 activation and differentiation, as well as IFN signaling (Fig. 6a, and data not shown). More precisely, the expression of genes (e.g., interferon regulatory factor 1 (IRF1) and proteasome subunit beta type-8 (PSMB8)) linked to STAT1 signaling,^40,41^ as well as the expression of STAT1 itself, was upregulated in naive T cells cultured with IFNγ-pretreated KCs (Fig. 6a). Notably, this link to IFNγ signaling was not due to IFNγ supplementation, since the IFNγ used for KC pretreatment was washed out before naive T cells were added to the KCs (Fig. S4B). Consistent with our findings described above, the expression of IRF1 and PSMB8 was significantly diminished, when CD58/CD2 signaling was blocked by downmodulation of CD2 expression on naive T cells, prior to coculture with IFNγ-pretreated KCs (Fig. 6b).

To further assess the role of CD58/CD2-mediated costimulation in KC-dependent T cell activation and STAT1 signaling in an independent experiment, we analyzed the phosphorylation, which reflects activation of STAT1 and other STAT molecules linked to Th1 and Th17 differentiation in T cells cocultured with untreated or IFNγ-pretreated KCs for 24 h. Phospho-flow cytometry revealed that STAT1 was highly phosphorylated in the naive T cells cultured with IFNγ-pretreated KCs (Fig. 6c). In addition, coculture with IFNγ-pretreated KCs led to enhanced phosphorylation of STAT5 (associated with Th1 differentiation) (Fig. S6B) and STAT3 (associated with Th17 differentiation) (Fig. 6d) in naive T cells. In contrast, the phosphorylation of STAT4, which is also linked to Th1 differentiation, was not enhanced in the naive T cells cultured with IFNγ-pretreated KCs compared with those cultured with untreated KCs (Fig. S6C). The phosphorylation of STAT1 but not that of STAT5 was significantly reduced upon blockade of CD58 and CD2 (Fig. 6c, S6B). In line with the previous observation that Th17 differentiation was not linked to CD58/CD2-mediated costimulation, enhanced STAT3 phosphorylation was not diminished upon blocking CD58/CD2-mediated costimulation (Fig. 6d).

To validate the link between CD58/CD2-dependent STAT1 phosphorylation and CD58/CD2-induced IFNγ production, we blocked STAT1 phosphorylation with fludarabine and analyzed IFNγ production in a coculture of IFNγ-pretreated KCs and naive T cells after 24 h. Phospho-flow cytometry revealed selective inhibition of KC-initiated STAT1 phosphorylation upon fludarabine pretreatment (Fig. 6e), whereas STAT3 and STAT5 phosphorylation was not significantly influenced (Fig. 6f, S6D). Indeed, this inhibition of KC-initiated STAT1 phosphorylation caused diminished IFNγ secretion (Fig. 6g). In contrast, the secretion of IL-2, which is not regulated by STAT1, was not affected by the inhibition of STAT1 phosphorylation (Fig. S6E).

Together, these experiments demonstrate that the costimulation of primary human naive T cells by antigen-loaded primary KCs through CD58/CD2 plays a crucial role in the differentiation of Th1 cells. Thus, KCs initiate STAT1 phosphorylation and subsequent IFNγ production in T cells.

### T cells located in the epidermis of psoriatic lesions express CD2 but not CD28

**Fig. 7:**
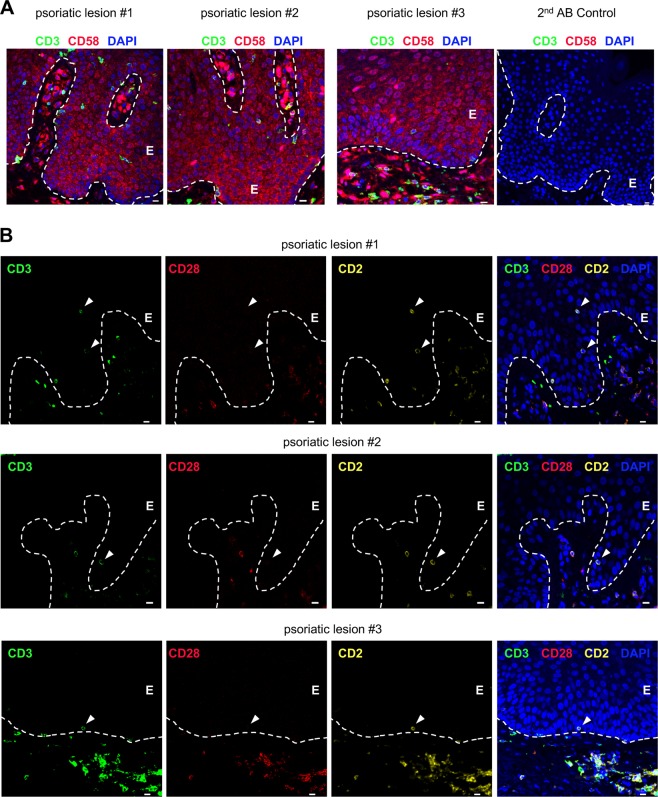
CD58 (on KCs) and CD2 (on T cells), not CD28 (on T cells), are detectable in the epidermis of psoriatic skin lesions. Representative immunohistochemical staining of punch biopsies of skin lesions from three different psoriasis patients, using an Opal-4-color IHC kit. **a** CD3 (green), CD58 (red), and DAPI (blue). For the secondary antibody (2nd AB) control, all reagents except the primary antibodies (αCD3 and αCD58) were used. **b** CD3 (green), CD28 (red), CD2 (yellow), and DAPI (blue). The dashed white line represents the border between the epidermis (E) and the dermis or a hair follicle. White triangles highlight epidermal T cells expressing CD2 but not CD28

To address the potential relevance of the interplay between T cells and KCs during the pathogenesis of chronic inflammatory skin diseases, we next analyzed skin tissue sections from psoriasis patients by using Opal Multiplex immunohistochemistry. To validate the potential activation of T cells in the skin through the CD58/CD2 interaction, we first determined the expression of CD58 on KCs in psoriatic skin lesions. Indeed, all KCs expressed CD58. Some individual cells in the epidermis and dermis showed even stronger expression levels (Fig. 7a). Analyzing CD2, the counterpart of CD58 expressed on T cells, in these skin lesions in comparison with CD28, the counterpart of CD80/86, interestingly revealed that all epidermal T cells expressed CD2 but not CD28 (Fig. 7b (white triangle)). In contrast, the T cells located in the dermis or hair follicle regions expressed both costimulatory molecules. The lack of CD80/86 on KCs and CD28 on T cells in situ in the epidermis supports our hypothesis that the interplay between CD58 and CD2 plays a crucial role in KC-mediated T cell costimulation and activation in the epidermis.

### Naive T cells lose CD45RA and CCR7 surface expression but upregulate the expression of skin-homing factors after direct interaction with IFNγ-pretreated keratinocytes

**Fig. 8:**
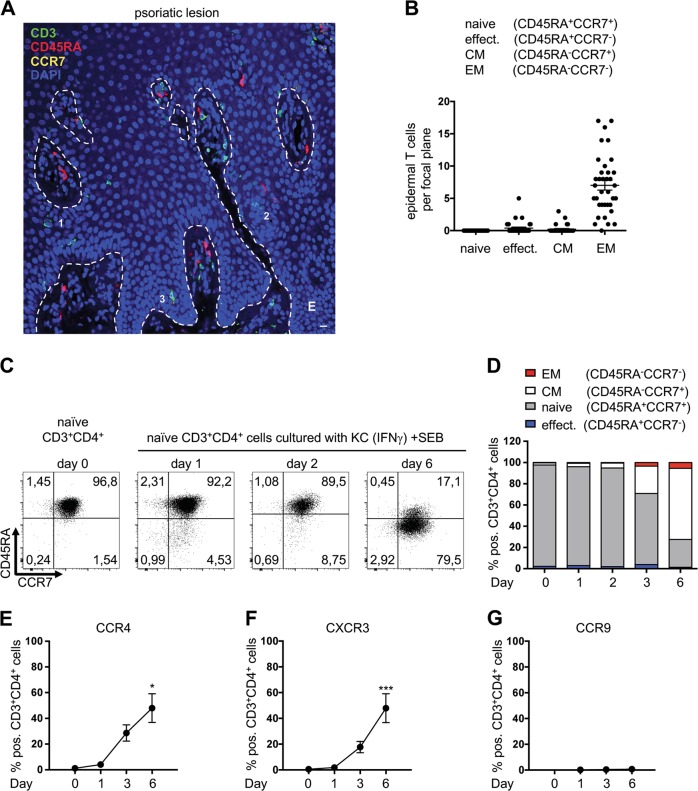
CD45RA^+^CCR7^−^ (effector), CD45RA^+^CCR7^−^ (CM), and CD45RA^−^CCR7^−^ (EM) T cells but not CD45RA^+^CCR7^+^ (naive) T cells are detected in psoriatic skin. Punch biopsies of psoriatic skin were stained for naive, effector (effect.), central memory (CM), and effector memory (EM) T cells. Naive T cells were cultured for 6 days with IFNγ-pretreated SEB-loaded KCs and analyzed for surface expression of the indicated chemokine receptors. **a** Representative immunohistochemical staining of a skin lesion from a psoriasis patient (punch biopsy), using an Opal-4-color IHC kit (CD3 (green), CD45RA (red), CCR7 (yellow), and DAPI (blue)). Cells 1 and 2: CD3^+^CD45RA^−^CCR7^−^ (EM); Cell 3: CD3^+^CD45RA^−^CCR7^+^ (CM). The dashed white line represents the border between the epidermis (E) and the dermis or a hair follicle. An example of single staining is shown in Figure S7A. **b** Statistical analysis of the number of epidermal T cells per focal plane, with the T cells separated into naive, effector, CM, and EM T cells. **c**–**d** Representative dot plot (**c**) and statistical evaluation (**d**) of the surface expression of CD45RA and CCR7 following coculturing of naive CD4^+^ T cells with IFNγ-pretreated KCs over time (*n* = 4 individual T cell donors). Effects of IFNγ-pretreated KCs on the surface expression of CCR4 (**e**), CXCR3 (**f**), and CCR9 (**g**) on naive T cells over time (*n* = 4 individual T cell donors). Data are represented as the mean ± SEM. ***p < 0.001 and *p < 0.05. See also Fig. S7

One remaining question was whether the phenotype of T cells in psoriatic skin lesions reflects the phenotype observed after costimulation of human naive T cells by IFNγ-pretreated KCs. In agreement with previous studies,^42,43^ immunofluorescence staining revealed the appearance of CD3^+^CD45RA^+^CCR7^−^ (effector), CD3^+^CD45RA^−^CCR7^+^ (CM), and CD3^+^CD45RA^−^CCR7^−^ (EM) T cells but not CD3^+^CD45RA^+^CCR7^+^ (naive) T cells within the dermis or epidermis of lesional skin (Fig. 8a, b (single-staining images in S7A)) and healthy skin (Fig. S7A). In particular, the T cells in the epidermis of psoriatic lesions showed no expression of CD45RA or CCR7 (Fig. 8a, b).

This finding may be explained by the results from our in vitro coculture system. In that system, T cells that encountered primary KCs were quickly activated and downregulated the expression of surface markers (CD45RA and CCR7) for naive T cells (Fig. 8c, d).

Interestingly, at the same time, naive T cells that were cultured with IFNγ-pretreated KCs specifically upregulated the expression of the skin-homing factors CCR4 (Fig. 8e) and CCR8 (Figure S7B). Together with these specific skin-homing factors, CXCR3 (Fig. 8f), CCR3 (Fig. S7C), and CCR5 (Fig. S7D) also exhibited upregulated expression. CXCR3 is a chemokine receptor that is found on T cells localized in different tissue types, including the skin. In contrast, the expression of CCR9 (Fig. 8g), which is associated with gut homing in T cells, was not upregulated. Thus, primary KCs selectively initiated the expression of skin-homing factors needed to retain T cells in the skin, whereby the characteristic expression profile of naive T cells was lost.

## Discussion

In this study, we aimed to clarify the potential of primary human KCs to act as nonprofessional APCs. In contrast to previous studies, our study used cocultures of primary human KCs with naive human T cells in the absence of any pAPCs (e.g., monocytes or DCs). Therefore, we demonstrated the ability of primary human IFNγ-pretreated KCs to costimulate and activate naive human T cells, even in the absence of CD80- or CD86-mediated costimulation. Instead, two other costimulatory receptor pairs were found to be involved in KC-dependent T cell activation, namely, CD58/CD2 and CD54/LFA-1.

In addition to influencing early T cell activation events, CD58/CD2 interactions played a crucial role during KC-initiated T cell differentiation. Upon blockade of CD58/CD2-mediated costimulation, inhibited IFNγ production led to a dysregulated Th1-associated cytokine milieu that caused severely diminished Th1 differentiation. Furthermore, STAT1 mRNA expression levels and phosphorylation in T cells revealed a close correlation to CD58/CD2-mediated costimulation. In an earlier study, CD2 signaling was found to enhance binding of STAT1 to intronic STAT binding regions in the IFNγ-encoding gene within lamina propria T cells.^44^ We observed diminished IFNγ production after the inhibition of STAT1 phosphorylation. Therefore, we propose that CD58/CD2-mediated costimulation of untransformed naive T cells by KCs initiates IFNγ production through STAT1 signaling.

In addition to the costimulation delivered through CD58/CD2, the interplay between CD54/LFA-1 was highly involved in the KC-dependent activation of naive T cells. Contrary to the effect of inhibiting CD58/CD2 signaling, the main effect of blocking, the CD54/LFA-1 interaction appeared to be the disruption of firm cell–cell contact formation, which also prevented costimulation through CD58/CD2 (Figure S3A, B). In contrast, adhesion of T cells to KCs was not influenced by blocking the interaction of CD58/CD2 (Figure S3A, B). This adhesion still allowed signaling via CD54/LFA-1 while costimulation through CD58/CD2 was inhibited. Verma et al. and others demonstrated that LFA-1 signaling promoted Th1 differentiation by upregulating T-bet expression.^45–48^ This result would explain the high expression of T-bet in naive T cells cultured with IFNγ-pretreated KCs in the presence of CD58/CD2 blockade (Figure S5C). In addition, another study showed that the interaction of CD54/LFA-1 increased the phosphorylation of STAT3 and inhibited differentiation into Th2 cells.^49,50^ STAT3 is a signaling molecule that regulates both T cell migration and Th17 differentiation.^51^ These observations agree with our data showing that KCs initiate Th17 differentiation of naive T cells independent of the interaction between CD58 and CD2. Furthermore, the CD54/LFA-1-mediated phosphorylation of STAT3 fits our observation that STAT3 phosphorylation is still upregulated in naive T cells cultured with IFNγ-pretreated KCs in the absence of CD58/CD2-mediated costimulation. Other studies also provided evidence for the involvement of LFA-1 in Th17 differentiation.^52^ However, the abolished T cell adhesion to KCs in our in vitro coculture system upon CD54/LFA-1 blockade did not allow further detailed analysis.

In our experiments, TGFβ, which is reported to be important in inducing Th17 differentiation (reviewed by Zhang et al.^38^), was produced by T cells cultured with IFNγ-pretreated KCs (Figure S5B). In combination with IL-6, small amounts of TGFβ are sufficient to trigger Th17 differentiation.^53^ The fact that the production of both cytokines was not related to CD58/CD2 interactions provides an additional explanation for the finding that Th17 polarization was CD58/CD2-independent.

Our coculture settings (i.e., IFNγ to stimulate KCs and an *S. aureus*-derived superantigen (SEB) for polyclonal T cell activation) were originally chosen to represent some of the pathophysiological conditions of psoriasis vulgaris. First, the preincubation of KCs with IFNγ mimics the situation of a proinflammatory environment. Thus, skin trauma, a trigger of the Koebner phenomenon of psoriasis, leads to the increase in IFNγ needed for KC activation.^54^ During wound healing, natural killer T cells (NKT cells) enter the wound and release IFNγ in early-wound infiltrates.^55,56^ Furthermore, colonization of the skin by SAg-producing *S. aureus* strains is associated with the severity of psoriasis vulgaris.^57^ In addition to these conditions found in psoriatic lesions, we mimicked, to some extent, the pathophysiological conditions of GvHD since we used alloreactive T cells in our coculture studies.

We assume that the described interaction of naive T cells with KCs under proinflammatory conditions may occur at the very early onset of inflammatory skin disease. A skin injury that destroys the barrier function of the basal membrane would explain how naive T cells could come into direct contact with KCs without prior T cell immigration into the skin. Then, naive T cells could be activated by KCs, differentiate into distinct T cell subsets, and upregulate skin-homing factors, enabling their retention in the skin. Consequently, the accumulation of Th1 and Th17 cells in skin lesions could be explained not only by the attraction of these T cell subsets through chemokines but also by KC-dependent T cell polarization through direct and indirect interactions in the skin.

Furthermore, if it is assumed that this process occurs during the onset of inflammatory skin diseases, we would not expect naive T cells to be present in the skin lesions of psoriatic patients, as this accumulation represents a late point during the pathogenesis of psoriasis. Direct interaction with KCs would trigger a change in the surface expression of chemokine receptors and provide a new explanation for the missing expression of surface markers for naive T cells on skin T cells.

Given the necessity of costimulation not only for the activation of naive T cells but also for the reactivation of memory T cells (reviewed by van der Heide et al.^58,59^), skin-resident memory T cells may also be regulated by costimulation through KCs. We observed that T cells located in the epidermis, which are mainly effector memory T cells, expressed CD2 but not CD28, whereas dermal T cells expressed both costimulatory molecules. These observations fit with the high expression of CD58 but lack of CD80 and CD86, the corresponding costimulatory receptors, on KCs. Interestingly, a study by Leitner et al. demonstrated the necessity of CD58/CD2-mediated costimulation for the activation and expansion of CD28^−^CD8^+^ T cells.^60^ In other inflammatory diseases, these CD3^+^CD28^–^ T cells tend to secrete proinflammatory cytokines, e.g., IFNγ and TNFα.^61–63^ Together with the exclusive location of CD28^−^ T cells in the epidermis of inflamed tissue, our study underlines the hypothesis that KCs activate skin-resident or injury-derived skin T cells through the interaction between CD58 and CD2, leading to a proinflammatory phenotype.

In the past, several systemically applied therapies based on biological agents targeting costimulation have been successful in treating moderate-to-severe psoriasis, e.g., efalizumab (a blocking antibody against LFA-1 (αCD11a, alpha subunit)) and alefacept (a dimeric fusion protein of the CD2-binding domain of CD58 (LFA-3)).^64,65^ However, treatment with efalizumab increases the risks of progressive multifocal leukoencephalopathy and lymphopenia, which ultimately led to its withdrawal in Europe and the USA.^66,67^ Alefacept was not approved in Europe because of its relatively low efficiency compared with other approved therapies.

To manage chronic GvHD, blocking agents against the most potent T cell costimulation pathway (CD80/CD86), namely, abatacept and belatacept, have been used.^68,69^ However, targeting CD80/CD86 may lead to undesired inhibition of coinhibitory pathways. Consequently, current therapeutics that solely block CD28 are undergoing testing.^70^ Interestingly, the first clinical trial using alefacept to control steroid-resistant GvHD has also proved its efficacy in this disease.^71–73^

In conclusion, our results reveal that KCs transmit signals through the costimulatory receptors CD58/CD2 and CD54/LFA-1, generating a micromilieu that enables Th1 and Th17 polarization independent of the presence of DCs. Consequently, future opportunities for more effective therapies treating chronic inflammatory skin diseases may lie in approaches that modulate KC-mediated activation of T cells directly in the skin rather than blocking the activation of T cells systemically. Notably, the perspective that a skin-directed blockade of costimulatory signal 2, even in the presence of signal 1, can induce antigen-specific tolerance (e.g., to KC antigens) opens up the possibility of inducing a long-lasting effect without the necessity of daily treatments. Finally, topical blockade of the costimulatory receptors LFA-1 and CD2 in the epidermis may even be superior to systemic treatment, as it is likely to minimize side effects.

## Materials and methods

### Primary cells and cell lines

Primary normal human epidermal keratinocytes (named primary human KCs in the paper) from the juvenile foreskin of a single donor were purchased from Promocell. KCs were cultured in serum-free medium (keratinocyte growth medium 2 (KGM-2), Promocell, Heidelberg). In some experiments, KCs were treated overnight with 100 ng/mL IFNγ (BioLegend, San Diego, CA) (as indicated in the respective figures).

Raji cells (ATCC, Manassas, CCL-86; Burkitt’s lymphoma cell line) were used as pAPCs. Raji cells were cultured in RPMI1640 medium (Gibco, Thermo Fisher Scientific, Waltham, MA) + 10 % FBS (PAN-BioTech, Aidenbach).

PBMCs were obtained from heparinized blood from healthy donors using Ficoll-Hypaque (Linaris, Dossenheim) density-gradient centrifugation. PBTs or naive CD4^+^ T cells were isolated from PBMCs using a Pan-T-cell isolation kit or naive CD4^+^ T-cell isolation kit (Miltenyi Biotec, Bergisch Gladbach). This study was approved by the Ethics Committee of Heidelberg University (S-089/2015).

### Keratinocyte/T cell coculture

KCs, either untreated or incubated with 100 ng/mL IFNγ overnight, were cultured with 5 µg/mL *S. aureus*-derived enterotoxin B (SEB) (Sigma Aldrich, St. Louis, MO) for 1 h at 37 °C and 5 % CO_2_. The KCs were washed three times with KGM-2 before PBTs or naive T cells were added in serum-free medium (XVIVO-15, Lonza, Basel). The cocultures were incubated for 24 h at 37 °C and 5 % CO_2,_ followed by analysis of the surface expression of the activation markers CD25 and CD69 by flow cytometry.

### T cell proliferation assay

To assess T cell proliferation, PBTs or naive T cells were stained with 1 µM 5-carboxyfluorescein diacetate (CFDA) (Thermo Fisher Scientific) for 20 min at 37 °C and 5% CO_2_ before they were cultured in serum-free medium (XVIVO-15, Lonza, Basel) with KCs. The cocultures were incubated for 72 h at 37 °C and 5% CO_2_, and T cell proliferation was analyzed by monitoring the dilution of CFDA by flow cytometry.

### Cytokine secretion assay

To assess the quantities of secreted cytokines, the supernatant of a coculture of naive T cells and KCs was collected, and 13 cytokines were analyzed by the LEGENDplex Human Th Panel (BioLegend, San Diego, CA) according to the manufacturer’s protocol.

### T cell differentiation assay

To assess T cell differentiation, naive T cells were cultured in serum-free medium (XVIVO-15, Lonza, Basel) with KCs. As a control, Raji cells were pretreated for 2 h with mitomycin C (20 µg/mL; Sigma Aldrich, St. Louis, MO) before naive T cells were added in serum-free medium (XVIVO-15, Lonza, Basel).

The cocultures were incubated for 72 h at 37 °C and 5% CO_2_. On day 3, fresh serum-free medium was added. On day 6, T cell differentiation was assessed by staining for transcription factors or cytokines after restimulation with phorbol 12-myristate 13-acetate (PMA) and ionomycin.

### PMA/ionomycin stimulation of differentiated T cells

To induce cytokine production, T cells cultured for 6 days with KCs were stimulated in a new well with 50 ng/mL PMA (Sigma Aldrich, St. Louis, MO) and 500 ng/mL ionomycin (Sigma Aldrich, St. Louis, MO) in the presence of 1 µg/mL GolgiStop (BD Bioscience, Franklin Lakes, NJ) for 4–6 h at 37 °C and 5% CO_2_. After stimulation, the cells were stained for surface markers and intracellular cytokines and analyzed by flow cytometry.

### Intracellular staining for cytokines and transcription factors

Cells that were already stained for surface markers were fixed with 1.5% PFA (Sigma Aldrich, St. Louis, MO) for 10 min at room temperature. Intracellular staining was performed in FACS buffer (PBS, 0.5% albumin Fraction V (Roth, Karlsruhe), and 0.1% NaN_3_) containing 0.1% saponin (Sigma Aldrich, St. Louis, MO). The cells were washed with the same buffer and resuspended in PBS for measurement.

For staining of transcription factors in differentiated T cells, surface marker staining was performed for 10 min on ice before the cells were fixed with True-Nuclear 1× Fix Concentration (BioLegend, San Diego, CA) for 1 h at room temperature. The T cells were permeabilized and stained for transcription factors (T-bet (4B10), BioLegend, San Diego, CA; GATA-3 (16E10A23), BioLegend, San Diego, CA; RORγt (Q21–559), BioLegend, San Diego, CA) in True-Nuclear 1× Perm Buffer (BioLegend, San Diego, CA) for 30 min at room temperature.

### Phospho-flow cytometry

For staining of phosphorylated proteins, surface marker staining was performed for 10 min on ice before the cells were fixed with Cytofix Fixation buffer (BD Bioscience, Franklin Lakes, NJ) for 10 min at 37 °C and 5% CO_2_. The T cells were permeabilized with Phosphoflow Perm Buffer III (BD Bioscience, Franklin Lakes, NJ) for 30 min on ice before the cells were stained with phospho-specific antibodies (p_Ser727_STAT1 (A15158B), BioLegend, San Diego, CA; p_Tyr705_STAT3 (13A3-1), BioLegend, San Diego, CA; p_Tyr693_STAT4 (38/p-stat4), BD Bioscience, Franklin Lakes, NJ; p_Tyr705_STAT5 (47/stat5), BioLegend, San Diego, CA; and p_Tyr641_STAT6 (A15137E), BioLegend, San Diego, CA).

### Phenotyping keratinocytes

Adherent KCs that were either left untreated or incubated with 100 ng/mL IFNγ (BioLegend, San Diego, CA) overnight were detached with warm trypsin/EDTA (Gibco, Thermo Fisher Scientific, Waltham, MA). The reaction was stopped by adding RPMI1640 medium + 10% FBS. Surface expression of HLA-DQ, HLA-DR, HLA-ABC and costimulatory receptors (CD40, CD54, CD58, CD80, CD86, and CD166) was analyzed by flow cytometry. The antibodies used are listed in the supplementary information.

### Antibody-mediated blocking of costimulatory molecules

In some coculture experiments, blocking antibodies (final concentration: 10 µg/mL) against CD54 (HCD54) (BioLegend, San Diego, CA), CD58 (TS2/9) (BioLegend, San Diego, CA), CD40 (5C3) (BioLegend, San Diego, CA), and CD40L (24–31) (BioLegend, San Diego, CA) and an isotype control (T8E5) (InVivoGen, San Diego, CA) were added to KCs cocultured with naive T cells in serum-free medium (XVIVO-15, Lonza, Basel). T cell activation was assessed after 24 h by analyzing the expression of the activation markers CD25 and CD69.

### CD2 downmodulation on naive T cells

Naive T cells were incubated with an anti-human CD2 antibody (IgM, Clone 2S5AE4; final concentration 0.04 mg/mL) overnight at 37 °C and 5% CO_2_ in RPMI1640 medium + 10% FBS.

### siRNA transfection of primary human keratinocytes

Adherent KCs were transfected with siRNA using GeneMute siRNA Transfection Reagent (SignaGen Laboratories, Rockville, MD). Briefly, 10 ng siRNA was mixed with GenMute reagent for 15 min before being added to KCs. After 24 h, the medium was replaced with fresh serum-free medium (KGM-2), and the KCs were cultured for 48 h at 37 °C and 5% CO_2_. In some experiments, the KCs were incubated overnight with 100 ng/mL IFNγ.

The following siRNAs were used: HLA-DRα siRNA (human; pool of three target-specific siRNAs, sc-37113, Santa Cruz Biotechnology, Dallas, TX), ICAM-1 siRNA (h) (pool of three target-specific siRNAs, sc-29354, Santa Cruz Biotechnology, Dallas, TX), and CD58 siRNA (h) (pool of three target-specific siRNAs, sc-42799, Santa Cruz Biotechnology, Dallas, TX) and Control siRNA-A (nontargeting siRNAs, sc-37007, Santa Cruz Biotechnology, Dallas, TX).

### Fludarabine treatment of naive T cells

Naive T cells were incubated with 100 µM fludarabine (Tocris, Bristol) or 0.1 % DMSO (Sigma Aldrich, St. Louis, MO) for 1 h at 37 °C and 5% CO_2_ in serum-free medium (XVIVO-15, Lonza, Basel). Subsequently, these pretreated or untreated naive T cells were added to IFNγ-pretreated SEB-loaded primary KCs and cocultured for 24 h at 37 °C and 5% CO_2_. Finally, secreted cytokines were analyzed by the cytokine secretion assay, and STAT phosphorylation was assessed by phospho-flow cytometry.

### Gene expression profiling

The nCounter Nanostring GX Human Immunology V2 panel (Nanostring, Seattle, WA) was used to analyze the expression of 579 immune and inflammation-associated target genes and 15 reference control genes in naive CD4^+^ T cells that were cultured for 4 h with either untreated or IFNγ-treated keratinocytes. Before RNA isolation, the T cells were isolated using flow cytometry. Total RNA was then extracted from 1 × 10^6^ naive T cells using the Direct-zol RNA Miniprep Kit (Zymo Research, Freiburg). All RNA samples were quantified by using a Qubit RNA assay kit (Thermo Fisher Scientific, Waltham, MA), and RNA integrity was assessed using an Agilent 2100 Bioanalyzer system.

A total of 25 µg total RNA (5 µL/sample) was mixed with nCounter® reporter CodeSet (3 µL) and nCounter^®^ capture ProbeSet (2 µL) with a hybridization buffer (5 µL) for an overnight hybridization reaction at 65 °C. The reaction was cooled to 4 °C, the samples were purified and immobilized on a cartridge, and the data were assessed on the nCounter SPRINT Profiler. The exported data were analyzed using Nanostring nSolver 4.0. A detailed description of the data analysis is described in the supplementary information.

### Immunofluorescence staining of human skin tissue

For immunohistochemistry (IHC) analysis, skin biopsies (5-mm punch biopsy) were collected from patients with plaque psoriasis. Skin was also taken from healthy volunteers. This study was approved by the Ethics Committee of Heidelberg University (S-392/2010).

Fluorescence staining of human skin tissue (5-mm biopsies) was performed using the Opal-4-Color Manual IHC Kit (PerkinElmer, Waltham, MA). In brief, formalin-fixed, paraffin-embedded tissue sections (2 µm) were deparaffinized in a series of xylene solutions and rehydrated with decreasing concentrations of alcohol, followed by formaldehyde fixation. The slides were subjected to antigen retrieval using microwave treatment followed by cooling at room temperature. The tissue sections were blocked with Antibody Diluent/Block reagent (PerkinElmer, Waltham, MA) and incubated for 1 h with a primary antibody against CD3 (SP7, DCS Innovative Diagnostic-Systeme, Hamburg, dilution 1:200) in a humidified chamber. Opal polymer HRP Ms + Rb (PerkinElmer, Waltham, MA) was used as a secondary antibody, followed by Opal signal generation using Opal 520 Fluorophore (PerkinElmer, Waltham, MA). The slides were placed in the antigen retrieval solution again and heated using microwave treatment to strip the primary–secondary–HRP complex. This procedure was repeated with primary antibodies against CD45RA (SPM504; Abcam, Cambridge; 1:100) (Opal 570 Fluorophore) and CCR7 (Y59; Abcam, Cambridge; 1:1000) (Opal 690 Fluorophore). Nuclei were stained using Roti-Mount FluorCare DAPI (Roth, Karlsruhe). As a negative control, immunofluorescence staining without primary antibodies was performed.

### Statistical analysis

Statistical analysis was performed with Prism 6 software (GraphPad, San Diego, CA). Values are expressed as the mean ± SEM. Analysis of variance (ANOVA) and an unpaired two-tailed Student’s *t* test were used to test for significant numerical differences among groups. Differences of *p* ≤ 0.05 were considered to be statistically significant (*p ≤ 0.05; **p ≤ 0.01; ***p ≤ 0.001; ****p < 0.0001).

## Supplementary information

Supplemental Figure

Supplemental Information

Supplemental Figurelegends
