# Supplementary material for: Keratinocytes costimulate naive human T cells via CD2: a potential target to prevent the development of proinflammatory Th1 cells in the skin
Source: Cell Mol Immunol. 2019 Jul 19;17(4):380–94. doi: 10.1038/s41423-019-0261-x (PMC7109061; doi:10.1038/s41423-019-0261-x)
Supplement: Supplementary file 1 — Supplemental Figure [file 41423_2019_261_MOESM1_ESM.pdf]

Figure S1

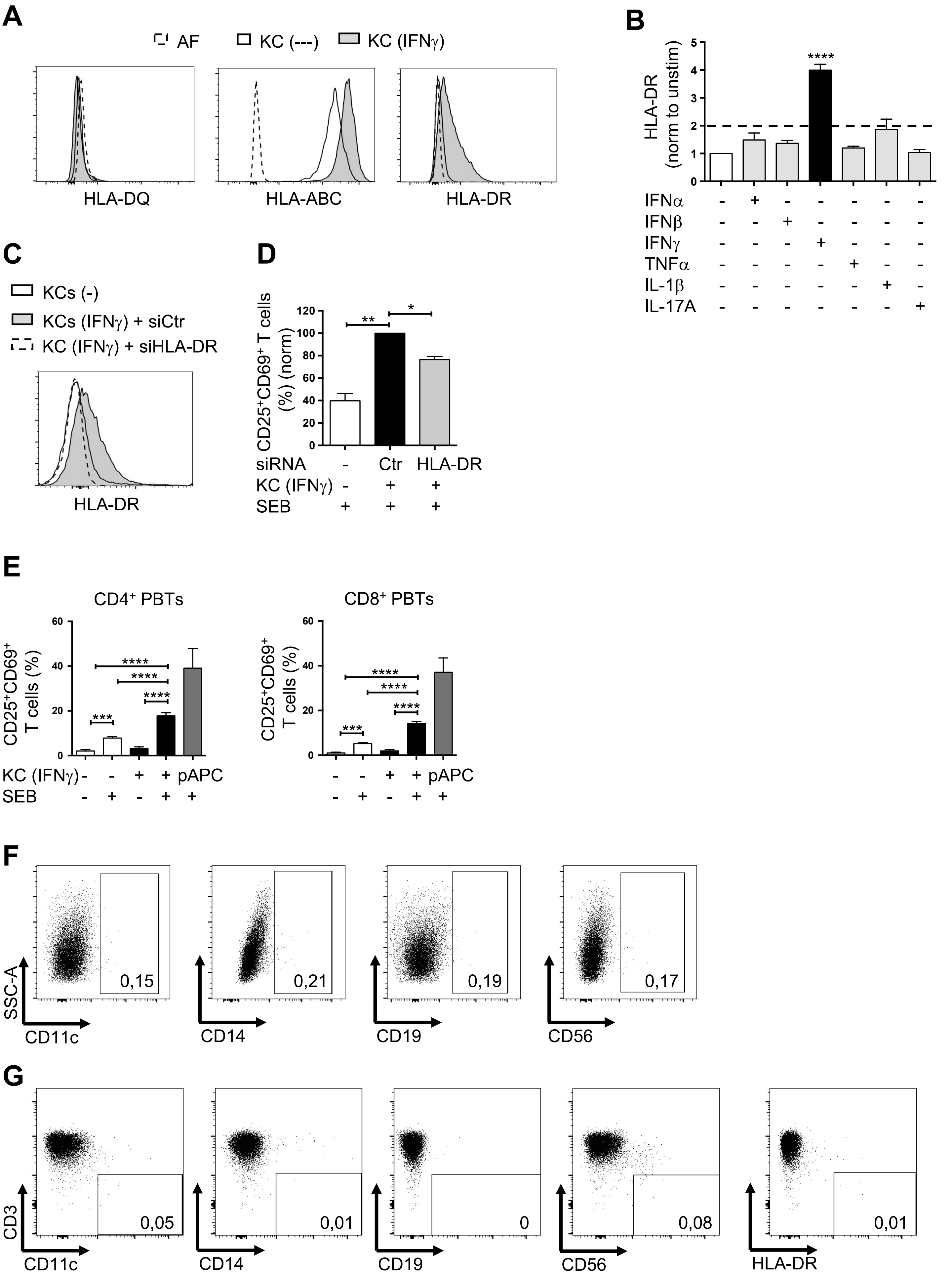
A
AF
KC (---)
KC (IFNγ)
HLA-DQ
HLA-ABC
HLA-DR
B
HLA-DR (norm to unstim)
****
IFNα
IFNβ
IFNγ
TNFα
IL-1β
IL-17A
C
KCs (-)
KCs (IFNγ) + siCtr
KC (IFNγ) + siHLA-DR
HLA-DR
D
CD25+CD69+ T cells (%) (norm)
siRNA - Ctr HLA-DR
KC (IFNγ) - + +
SEB + + +
E
CD4+ PBTs
CD8+ PBTs
CD25+CD69+ T cells (%)
KC (IFNγ) - - + + pAPC
SEB - + - + +
F
SSC-A
CD11c
CD14
CD19
CD56
0,15
0,21
0,19
0,17
G
CD3
CD11c
CD14
CD19
CD56
HLA-DR
0,05
0,01
0
0,08
0,01

Figure S2

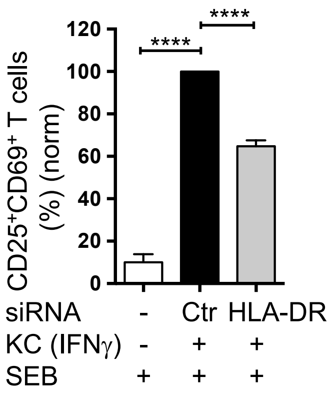
CD25+CD69+ T cells (%) (norm)
120
100
80
60
40
20
0
****
****
siRNA - Ctr HLA-DR
KC (IFNγ) - + +
SEB + + +

Figure S3

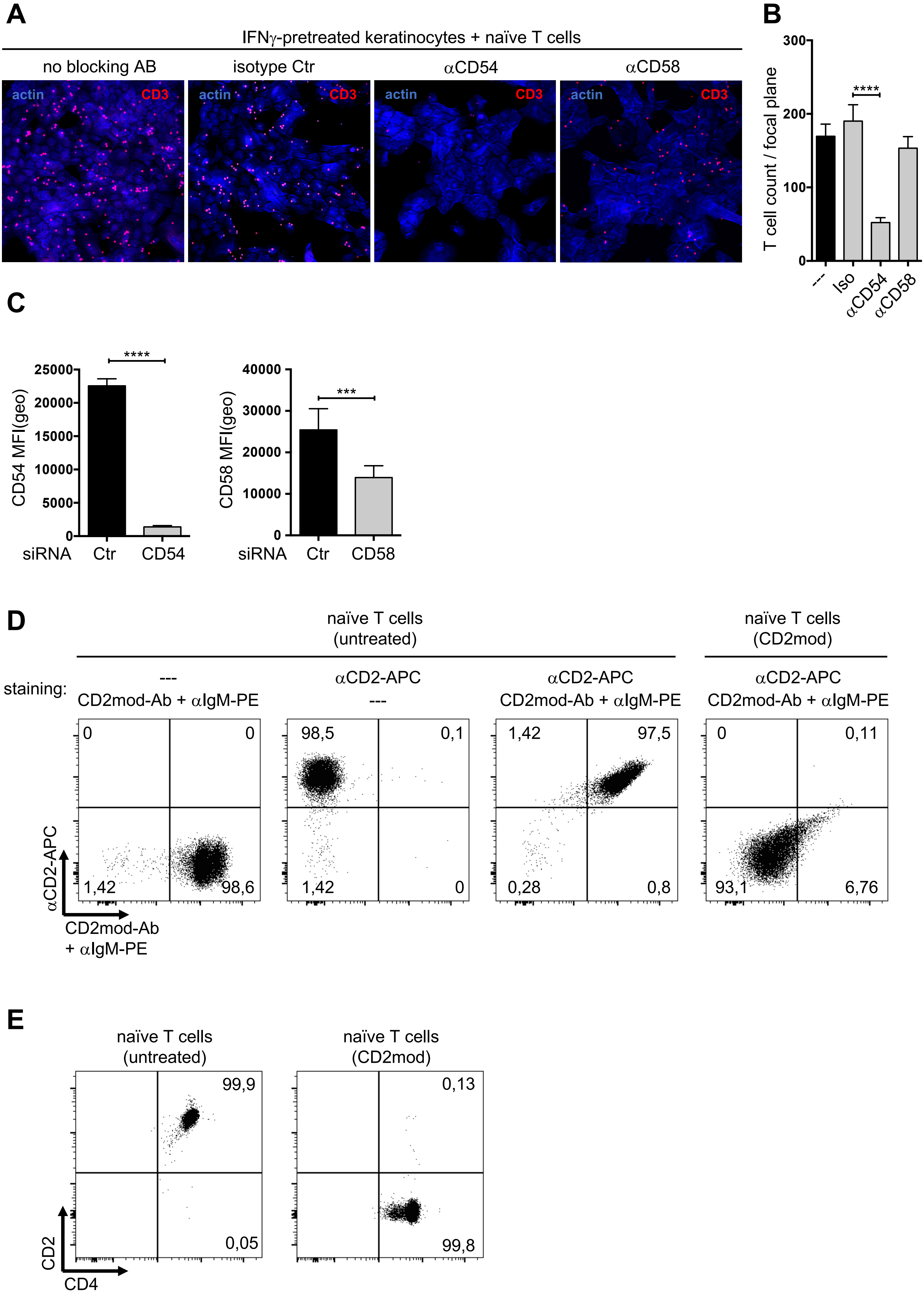

A
IFNγ-pretreated keratinocytes + naïve T cells
no blocking AB
isotype Ctr
αCD54
αCD58
actin
CD3
B
T cell count / focal plane
300
200
100
0
****

Iso
αCD54
αCD58
C
CD54 MFI(geo)
25000
20000
15000
10000
5000
0
****
siRNA
Ctr
CD54
CD58 MFI(geo)
40000
30000
20000
10000
0
***
siRNA
Ctr
CD58
D
naïve T cells (untreated)
naïve T cells (CD2mod)
staining:

CD2mod-Ab + αIgM-PE
αCD2-APC

αCD2-APC
CD2mod-Ab + αIgM-PE
αCD2-APC
CD2mod-Ab + αIgM-PE
0
0
1,42
98,6
98,5
0,1
1,42
0
1,42
97,5
0,28
0,8
0
0,11
93,1
6,76
αCD2-APC
CD2mod-Ab + αIgM-PE
E
naïve T cells (untreated)
naïve T cells (CD2mod)
99,9
0,05
0,13
99,8
CD2
CD4

Figure S4

A

	KC (-) -SEB	KC (-) +SEB	KC (IFNγ) +SEB	pAPC +SEB
**IL-5**	n.d.	n.d.	n.d.	n.d.
**IL-13**	<100 pg/mL	<100 pg/mL	<100 pg/mL	<100 pg/mL
**IL-2**	<100 pg/mL	533,8 pg/mL	3149 pg/mL	8473 pg/mL
**IL-6**	367,2 pg/mL	908,4 pg/mL	900,2 pg/mL	<100 pg/mL
**IL-9**	<100 pg/mL	<100 pg/mL	<100 pg/mL	<100 pg/mL
**IL-10**	<100 pg/mL	<100 pg/mL	141,5 pg/mL	175,5 pg/mL
**IFNg**	<100 pg/mL	4572,6 pg/mL	15389 pg/mL	15389 pg/mL
**TNFa**	<100 pg/mL	11,6 pg/mL	155,9 pg/mL	9512,74 pg/mL
**IL-17A**	n.d.	n.d.	n.d.	n.d.
**IL-17F**	n.d.	n.d.	n.d.	n.d.
**IL-4**	n.d.	n.d.	n.d.	n.d.
**IL-21**	n.d.	n.d.	n.d.	n.d.
**IL-22**	n.d.	n.d.	n.d.	n.d.

B

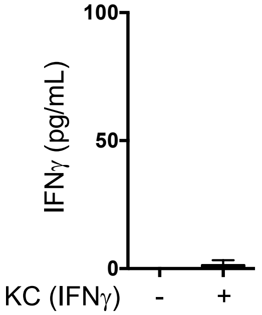

C

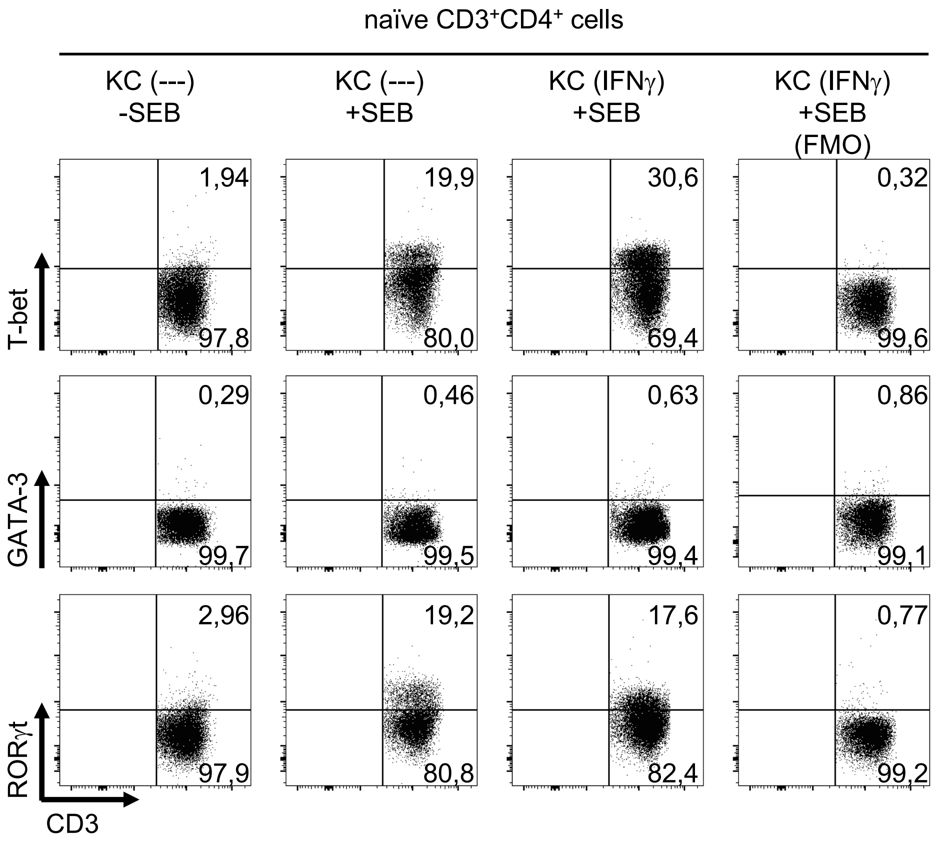

D

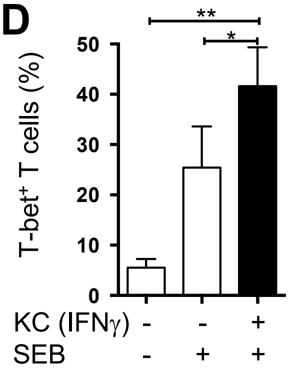

E

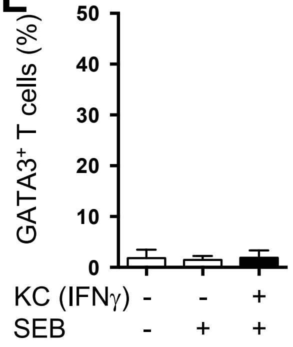

F

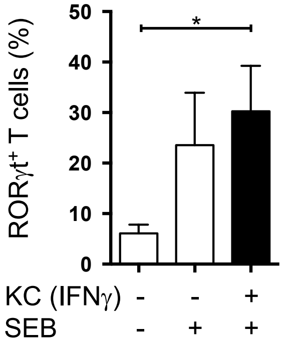

Figure S5

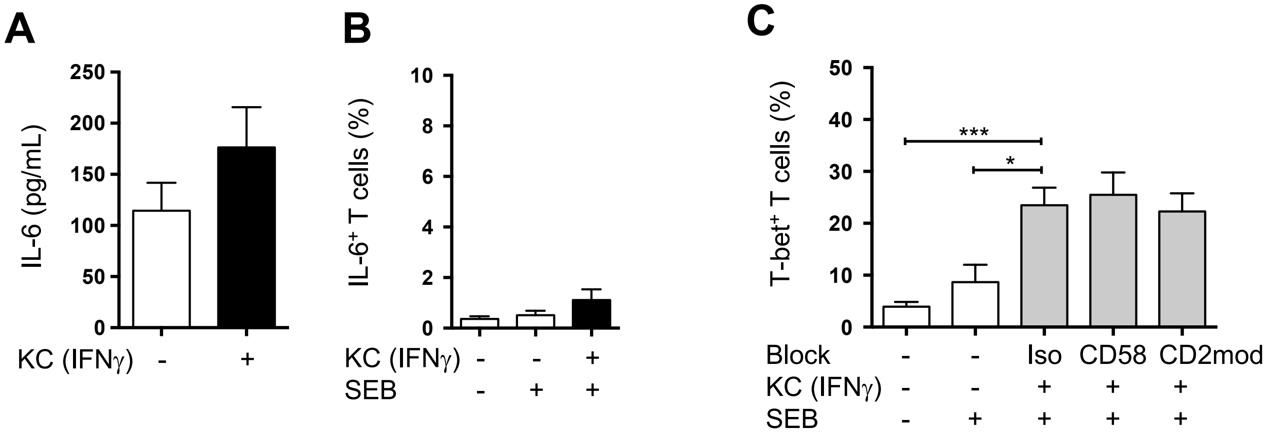
A
IL-6 (pg/mL)
250
200
150
100
50
0
KC (IFNγ) - +
B
IL-6+ T cells (%)
10
8
6
4
2
0
KC (IFNγ) - - +
SEB - + +
C
T-bet+ T cells (%)
50
40
30
20
10
0
***
*
Block - - Iso CD58 CD2mod
KC (IFNγ) - - + + +
SEB - + + + +

Figure S6

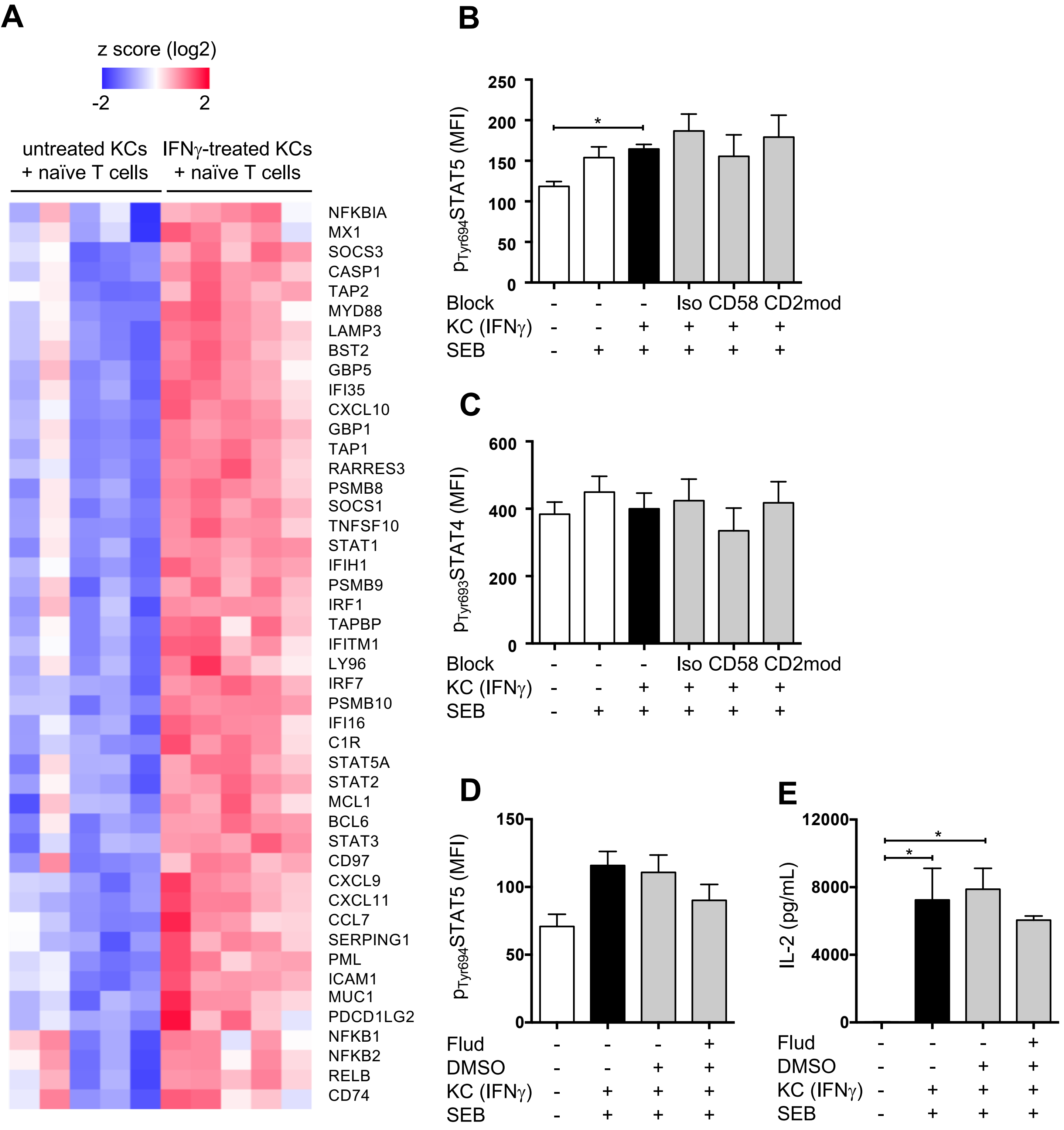

A
z score (log2)
-2
2
untreated KCs + naïve T cells
IFNγ-treated KCs + naïve T cells
NFKBIA
MX1
SOCS3
CASP1
TAP2
MYD88
LAMP3
BST2
GBP5
IFI35
CXCL10
GBP1
TAP1
RARRES3
PSMB8
SOCS1
TNFSF10
STAT1
IFIH1
PSMB9
IRF1
TAPBP
IFITM1
LY96
IRF7
PSMB10
IFI16
C1R
STAT5A
STAT2
MCL1
BCL6
STAT3
CD97
CXCL9
CXCL11
CCL7
SERPING1
PML
ICAM1
MUC1
PDCD1LG2
NFKB1
NFKB2
RELB
CD74
B
pTyr694STAT5 (MFI)
250
200
150
100
50
0
*
Block - - - Iso CD58 CD2mod
KC (IFNγ) - - + + + +
SEB - + + + + +
C
pTyr693STAT4 (MFI)
600
400
200
0
Block - - - Iso CD58 CD2mod
KC (IFNγ) - - + + + +
SEB - + + + + +
D
pTyr694STAT5 (MFI)
150
100
50
0
Flud - - - +
DMSO - - + +
KC (IFNγ) - + + +
SEB - + + +
E
IL-2 (pg/mL)
12000
8000
4000
0
*
*
Flud - - - +
DMSO - - + +
KC (IFNγ) - + + +
SEB - + + +

Figure S7

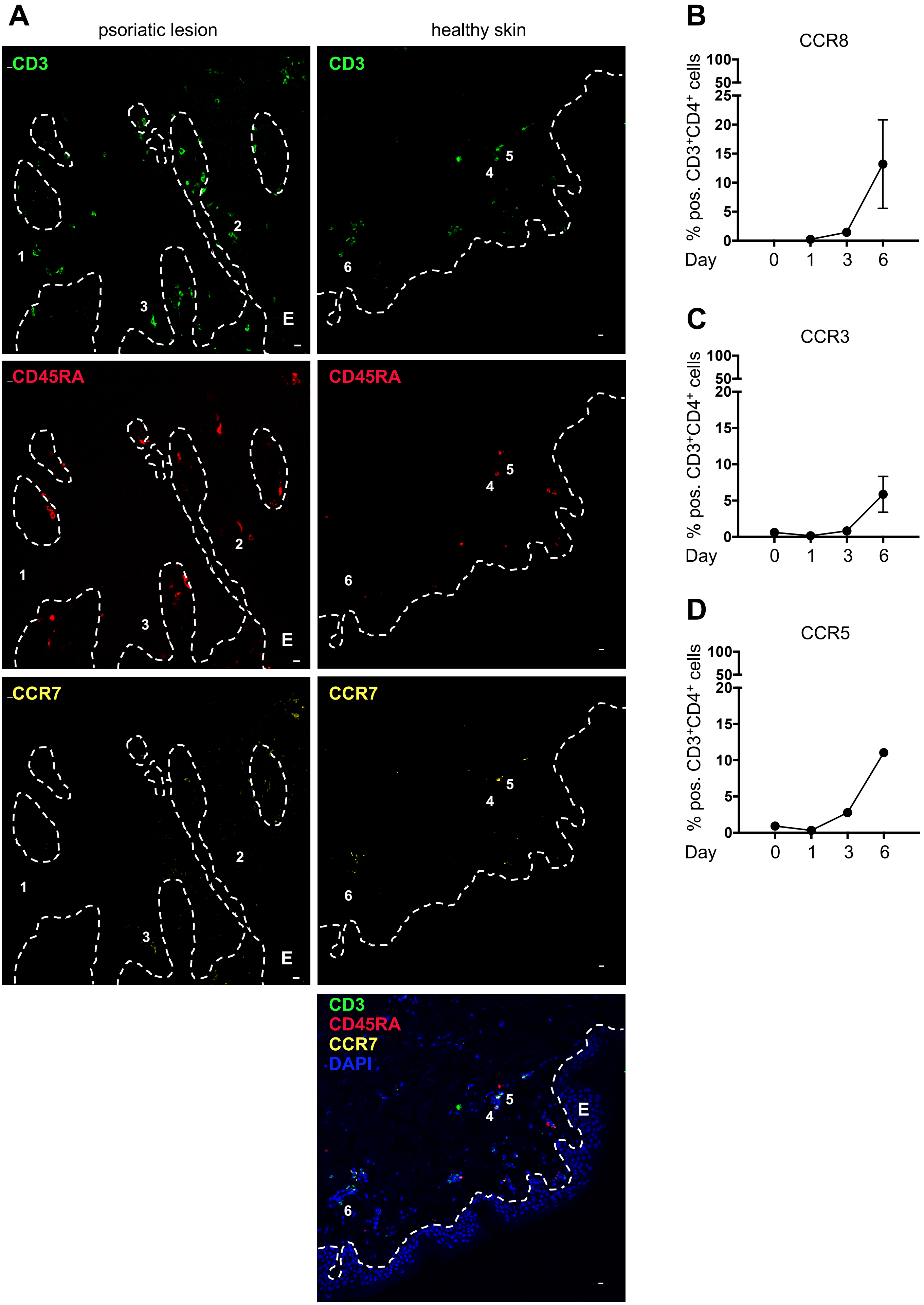
A
psoriatic lesion
healthy skin
CD3
CD45RA
CCR7
DAPI
1
2
3
4
5
6
E
B
CCR8
C
CCR3
D
CCR5
% pos. CD3+CD4+ cells
Day
0
1
3
6
100
50
25
20
15
10
5
0
