# Supplementary material for: Keratinocytes costimulate naive human T cells via CD2: a potential target to prevent the development of proinflammatory Th1 cells in the skin
Source: Cell Mol Immunol. 2019 Jul 19;17(4):380–94. doi: 10.1038/s41423-019-0261-x (PMC7109061; doi:10.1038/s41423-019-0261-x)
Supplement: Supplementary file 2 — Supplemental Information [file 41423_2019_261_MOESM2_ESM.docx]

# Supplemental Information

**Figure S1: siRNA-mediated knockdown of HLA-DR in primary human keratinocytes and phenotyping of primary human keratinocytes and peripheral blood T cells.**

**A-C:** Role of HLA-DR in KC-induced T cell activation. Representative histograms **(A)** of MHC I (HLA-ABC) and MHC II (HLA-DR, HLA-DQ) surface expression on untreated KCs (black line, white background) and IFNγ-pretreated KCs (black line, grey background) cultured for 24 h. Untreated KCs stained with isotype control antibody (AF; dashed line, white background). Statistical evaluation **(B)** of HLA-DR expression on primary KCs treated for 24 h with the indicated cytokines. Histograms **(C)** of HLA-DR expression on untreated KCs (black line, white background) and IFNγ-pretreated KCs incubated with control siRNA (siCtr; black line, grey background) or siRNA against HLA-DR (siHLA-DR; dashed line, white background). CD25 and CD69 expression **(D)** on T cells cultured for 24 h with siRNA-treated KCs.

**E:** Expression of CD25 and CD69 on CD4^+^ and CD8^+^ T cells cultured for 24 h with untreated KCs (white bars) or IFNγ-pretreated KCs (black bars) loaded with (+) or without (-) SEB and then analyzed by flow cytometry. Professional antigen-presenting cells (pAPCs) loaded with SEB served as positive control (grey bars) (n = 3 individual T cell donors). **F-G**: Phenotyping of primary human KCs and primary human PBT preparations. Representative dot plots of surface expression of CD11c, CD14, CD19 and CD56 expression on untreated KC **(F)** and surface expression of CD3, CD11c, CD14, CD19, CD56 and HLA-DR expression on cells of the PBT purification **(G).** Data is represented as mean ± SEM. **** = p<0.0001; *** = p<0.001; ** = p<0.01; * = p<0.05.

**Figure S2: Effect of HLA-DR knockdown on keratinocyte-mediated activation of naïve CD4^+^ T cells.**

Effect of siRNA-mediated HLA-DR knockdown in primary human KCs on the expression of CD25 and CD69 on naïve CD4^+^ T cells cultured for 24 h with either untreated KCs (white bars) or IFNγ-pretreated KCs (black and grey bars) loaded with SEB (n = 5 individual T cell donors). Expression was analyzed by flow cytometry. Data is represented as mean ± SEM. **** = p<0.0001.

**Figure S3: Effect of blocking antibodies against CD54 and CD58 on T cell adherence to IFNγ-pretreated keratinocytes and phenotyping of siRNA-treated keratinocytes and CD2-modulated T cells.**

**A-B:** Naïve CD4^+^ T cells were cultured for 4 h with IFNγ-pretreated KCs (black and grey bars) loaded with SEB in the presence of blocking antibodies (as indicated) and analyzed by confocal microscopy. **A:** Representative immunofluorescence staining for F-actin (blue) and CD3 (red) is shown. **B:** Statistical evaluation of the effect of isotype control antibodies (Iso) or blocking antibodies against costimulatory receptors (CD54, CD58) on the T cell adhesion to IFNγ-pretreated KCs (black bar (no antibody) and grey bars) was calculated as number of T cells per optical field (n = 6 individual T cell donors).

**C:** Surface expression of CD54 and CD58 on IFNγ-pretreated KCs 24 h after siRNA-treatment (control (Ctr, black bar), siCD54 (CD54, grey bar) and siCD58 (CD58, grey bar) (n ≥ 3 independent experiments). **D:** Representative dot plots of untreated or CD2-downmodulated (CD2mod) naïve T cells. Both untreated and CD2mod T cells, were stained with CD2 modulatory antibody (CD2mod-Ab) and anti-IgM-PE and/or APC-labeled anti-CD2 antibody (αCD2-APC) (as indicated).

**E:** Representative dot plots of surface expression of CD4 and CD2 on untreated and CD2mod naïve CD4^+^ T cells. Data is represented as mean ± SEM. **** = p<0.0001, *** = p<0.001.

**Figure S4: Cytokine secretion and expression of transcription factors in keratinocyte-T cell cocultures.**

Naïve CD4^+^ T cells were cultured for the indicated time points with untreated KCs or IFNγ-pretreated KCs loaded with (+) or without (-) SEB and then analyzed by flow cytometry. **A:** Mean values of the amounts of cytokines secreted into the supernatant after 24 h coculture (n = 6 individual T cell donors). pAPCs loaded with SEB cocultures with T cells served as positive control. Cytokine secretion into the supernatant was analyzed by cytokine bead array. Not detectable cytokines are indicated by ‘n.d.’. Cytokines with a concentration below 100 pg/mL were not considered for further analysis (<100 pg/mL). **B:** Statistical evaluation of the amount of secreted IFNγ in the supernatant of untreated KCs (white bar) or IFNγ-pretreated KCs (black bar). KCs were washed three times with KGM-2 before XVIVO-15 medium was added. After 4 h, IFNγ secretion was analyzed by cytokine bead array (n ≥ 3 independent experiments). **C-F:** Representative dot plots **(C)** and statistical evaluation of T-bet **(D)** expression, GATA3 **(E)** expression and RORγt **(F)** expression after 6 days coculture (n = 5 individual T cell donors). Intracellular transcription factor expression was analyzed after PMA/ionomycin treatment. Fluorescence minus one (FMO) of respective transcription factors were used to determine positive staining. Data is represented as mean ± SEM. ** = p<0.01; * = p<0.05.

**Figure S5: IL-6 production by primary human keratinocytes but not T cells and keratinocyte-mediated T-bet expression.**

**A:** Statistical evaluation of the amount of secreted IL-6 in the supernatant of untreated KCs (white bar) or IFNγ-pretreated KCs (black bar) after 24 h (n ≥ 3 independent experiments). Cytokine secretion into the supernatant was analyzed by cytokine bead array. **B:** Intracellular IL-6 staining in naïve CD4^+^ T cells cultured with untreated KCs (white bars) or IFNγ-pretreated KCs (black bars) (n ≥ 5 individual T cell donors). **C:** Effect of isotype control (Iso) and blocking antibodies against CD58 and CD2-downmodulation (CD2mod) on T cell-specific T-bet expression in a 6 days coculture with untreated KCs (white bars) or IFNγ-pretreated KCs (grey bars) (n = 5 individual T cell donors). Intracellular transcription factor expression was analyzed after PMA/ionomycin treatment. Data is represented as mean ± SEM. *** = p<0.001; * = p<0.05.

**Figure S6: mRNA expression profile and STAT4 phosphorylation during keratinocyte-dependent activation of naïve CD4^+^ T cells.**

Naïve CD4^+^ T cells were cultured for the indicated time points with untreated KCs or IFNγ-pretreated KCs loaded with SEB and then analyzed by Nanostring nCounter GEx (mRNA expression) or flow cytometry. **A:** Heat map of regulated genes in naïve CD4^+^ T cells in a 4 h coculture (each row of either ‘untreated KCs + naïve T cells’ or ‘IFNγ-pretreated + naïve T cells’ represents data points derived from T cells from one individual donor). mRNA expression was analyzed by Nanostring nCounter human Immunology Panel.

**B-C:** Effect of isotype control antibodies (Iso), blocking antibodies against CD58, or CD2-downmodulation (CD2mod) on T cell-specific STAT5 **(B)** and STAT4 **(C)** phosphorylation after 24 h coculture (n = 5 individual T cell donors). **D-E:** Effect of fludarabine (Flud) treatment on T cell-specific STAT5 **(E)** or IL-2 secretion into the supernatant **(E)** (n = 4 individual T cell donors) after 24 h coculture. Data is represented as mean ± SEM. * = p<0.05.

## Figure S7: Upregulated surface expression of skin-homing factors on naïve T cells cultured with IFNγ-pretreated keratinocytes.

Punch biopsies of psoriatic skin were stained for naïve, effector, central memory and effector memory T cells. Naïve T cells were cultured for 6 days with IFNγ-pretreated and SEB-loaded KCs and were analyzed for surface expression of the indicated chemokine receptors. **(A)** Representative immunohistochemistry staining of a skin lesion of a psoriasis patient or healthy donor (punch biopsy) using Opal-4-color IHC kit (CD3 (green), CD45RA (red), CCR7 (yellow), DAPI (blue)). Cell 1, 2, 6: (CD3^+^CD45RA^-^CCR7^-^ (EM); Cell 3, 5: (CD3^+^CD45RA^-^CCR7^+^ (CM)); Cell 4: (CD3^+^CD45RA^+^CCR7^-^ (effector)). The dashed white line represents the border of epidermis (E) to dermis or hair follicle, respectively. **B-D:** Effect of IFNγ-pretreated KCs on the surface expression of CCR8 **(B)**, CCR3 **(C)** and CCR5 **(D)** on naïve T cells over time (n = 4 individual T cell donors). Data is represented as mean ± SEM.

|  | surface expression on untreated KCs | surface expression on IFNγ-pretreated KCs | surface expression on pAPCs |
| --- | --- | --- | --- |
| CD48 | - | - | ++ |
| CD70 | - | - | +++ |
| CD137L | - | - | + |
| CD137 | - | - | ++ |
| CD252 | - | - | ++ |
| Galectin-9 | - | - | +++ |

**Table S1: Surface expression of costimulatory receptors on primary keratinocytes and professional antigen-presenting cells.**

Primary KCs were either pretreated with IFNγ for 24h or left untreated before surface expression of costimulatory receptors was analyzed. As positive control, pAPCs were used. Relative expression level was assessed by the geometric MFI (MFI geo). No expression: MFI geo < 10 ‘ - ‘. Low expression: MFI geo < 100 ‘ + ‘. Medium expression: 100 < MFI geo < 1000 ‘ ++ ‘. High expression: 1000 < MFI geo ‘ +++ ‘.

**Supplemental Experimental Procedures**

## Immunofluorescence Microscopy

KCs, either untreated or incubated with 100 ng/mL IFNγ overnight, were cultured on coverslips. These KCs were loaded with SEB for 1 h at 37 °C, 5 % CO_2_ and washed three times with serum-free KGM-2 before naïve T cells were added in serum-free medium (XVIVO-15, Lonza, Basel). After coincubation for different time periods (as indicated in the figures), cells were fixed with 1.5 % PFA for 20 min, permeabilized with 0.1 % saponin (in PBS + 10 % FBS) and stained with DAPI (Sigma Aldrich) (100 ng/mL), phalloidin-AF488 (Thermo Fisher Scientific, Waltham, Massachusetts) (0.4 U/mL), anti-phospho-L-plastin (UHZ) (Epitomics, Abcam, Cambridge, UK) (1 µg/mL) or anti-CD11a (LFA-1; Hl111) (Biolegend, San Diego, California) (2 µg/mL). Anti-rb-Cy3 (Dianova, Hamburg) (1 µg/mL) was used as secondary antibody. Laser scanning confocal microscopy was performed using a Nikon A1R (40x objective; NA = 1.3).

## Flow cytometry

Monoclonal antibodies recognizing the following surface markers and molecules were used for flow cytometry: CD2 (RPA-2.10), CD3 (SK7), CD4 (SK3), CD8 (SK1), CD25 (2A3), CD40 (5C3), CD45RA (HI30), CD69 (L78), CD54 (HA58), CD58 (TS2/9), CD80 (L307.4), CD86 (2331), CCR3 (5E8), CCR4 (L291H4), CCR5 (REA245), CCR6 (11A9), CCR7 (150503), CCR8 (L263G8), CCR9 (L053E8), CCR10 (6588-5), CXCR3 (G025H7), IL-2 (5344.111), IL-4 (301.211), IL-6 ( MQ2-13A5), IL-17A (SCPL1362), IFNγ (B27), TGFβ (TW4-9E7), HLA-DR (L243), HLA-ABC (W6/32), HLA-DQ (HLADQ1). 7AAD was used to discriminate dead cells. All antibodies were obtained from BD Bioscience (Franklin Lakes, New Jersey), eBioscience (Affymetrix, Santa Clara, California) or BioLegend (San Diego, California).

## nCounter Data analysis

The generated RCC-files were imported to NanoString nSolver 4.0 and the ‘raw-data’ was analyzed using the nCounter Advanced Analysis package (Version 2.0.115). This Analysis package includes automated normalization gene selection, experimental design quality control (QC), differential expression analysis and agglomerative clustering. Selected house-keeping genes (HKG), used as internal references genes are listed in table (Table HKGs).

| **Selected HKG** | **Accession number** |
| --- | --- |
| EEF1G | NM_001404.4:1150 |
| OAZ1 | NM_004152.2:313 |
| RPL19 | NM_000981.3:315 |
| POLR2A | NM_000937.2:3775 |
| TBP | NM_001172085.1:587 |
| PPIA | NM_021130.2:925 |
| GAPDH | NM_002046.3:972 |
| TUBB | NM_178014.2:320 |
| G6PD | NM_000402.2:1155 |
| HPRT1 | NM_000194.1:240 |
| POLR1B | NM_019014.3:3320 |
| SDHA | NM_004168.1:230 |

**Table S2: House-keeping of nCounter Analysis.**

Selected HKG which were used as internal references genes for nCounter Advanced Analysis.

In the following analysis, probes were called detected when they had more than double the counts of the median negative controls (median count: 14.5 counts). For differential expression (DE) analysis, genes were tested for DE in response to the selected covariate (untreated KCs + naïve T cells (1), IFNγ-pretreated KCs + naïve T cells (2), IFNγ-pretreated KCs + naïve T cells (CD2mod) (3); DE: (1) vs (2); (2) vs. (3)). To predict the expression of all genes, a single linear regression was fitted using all selected covariates. To estimate differential expression, either the mixed negative binomial model (Wald test), the simplified negative binomial model or the loglinear model was used. Gene expression is considered as significantly changed if the p-value is below 0.05.

For following pathway analysis, the DE of all detected probes was calculated and the fold-change (log-2 value) and the respective p-value (- log-10 value) were imported to Ingenuity Pathway Analysis (Qiagen, Hilden). Pathways were considered significantly regulated if the p-value of the DE of the assigned genes was below 0.05 (-log-10 ≥ 1.3).
