# Supplementary material for: Keratinocytes costimulate naive human T cells via CD2: a potential target to prevent the development of proinflammatory Th1 cells in the skin
Source: Cell Mol Immunol. 2019 Jul 19;17(4):380–94. doi: 10.1038/s41423-019-0261-x (PMC7109061; doi:10.1038/s41423-019-0261-x)
Supplement: Supplementary file 3 — Supplemental Figurelegends [file 41423_2019_261_MOESM3_ESM.pdf]

Supplemental Information

Figure S1: siRNA-mediated knock down of HLA-DR in primary human keratinocytes and phenotyping of primary human keratinocytes and PBTs.

A-C: Role of HLA-DR in KC-induced T cell activation. Representative histograms **(A)** of MHC I (HLA-ABC) and MHC II (HLA-DR, HLA-DQ) surface expression on untreated KCs (black line, white background) and IFNγ-pretreated KCs (black line, grey background) cultured for 24 h. Autofluorescence (AF; dashed line, white background). Histograms **(B)** of HLA-DR expression on untreated KCs (black line, white background) and IFNγ-pretreated KCs incubated with control siRNA (siCtr; black line, grey background) or siRNA against HLA-DR (siHLA-DR; dashed line, white background). CD25 and CD69 expression **(C)** on T cells cultured for 24 h with siRNA-treated KCs.

D: Expression of CD25 and CD69 on $CD4^+$ and $CD8^+$ T cells cultured for 24 h with untreated KCs (white bars) or IFNγ-pretreated KCs (black bars) loaded with (+) or without (-) SEB and then analyzed by flow cytometry. Professional antigen-presenting cells (pAPC) loaded with SEB served as positive control (n = 3 individual T cell donors).

E-F: Phenotyping of primary human keratinocytes and primary human PBT preparations. Representative dot plots of surface expression of CD11c, CD14, CD19 and CD56 expression on untreated KC **(E)** and surface expression of CD3, CD11c, CD14, CD19, CD56 and HLA-DR expression on cells of the PBT purification **(F).** Data is represented as mean ± SEM. ****=p<0.0001; ***=p<0.001; **=p<0.01; *=p<0.05.

Figure S2: Effect of HLA-DR knock down on keratinocyte-mediated activation of naïve $CD4^+$ T cells.

Effect of siRNA-mediated HLA-DR knock-down in primary human KCs on the expression of CD25 and CD69 on naïve $CD4^+$ T cells cultured for 24 h with either

untreated KCs (white bars) or IFNγ-pretreated KCs (black and grey bars) loaded with SEB (n = 5 individual T cell donors). Expression was analyzed by flow cytometry. Data is represented as mean ± SEM. ****=p<0.0001.

Figure S3: Effect of blocking antibodies against CD54 and CD58 on T cell adherence to IFNγ-pretreated keratinocytes and phenotyping of siRNA-treated keratinocytes and CD2 modulated T cells.

A-B: Naive $CD4^+$ T cells were cultured for 4 h with untreated KCs (white bars) or IFNγ-pretreated KCs (black and grey bars) loaded with SEB in the presence of blocking antibodies (as indicated) and analyzed by confocal microscopy. **A:** Representative immunofluorescence staining for F-actin (blue) and CD3 (red) is shown. **B:** Statistical evaluation of the effect of isotype control antibodies (Iso) or blocking antibodies against costimulatory receptors (CD54, CD58) on the T cell adhesion to IFNγ-pretreated KCs (black bar (no antibody) and grey bars) was calculated as number of T cells per optical field (n = 6 individual T cell donors).

C: Surface expression of CD54 and CD58 on IFNγ-pretreated KCs 24 h after siRNA-treatment (control (siCtr), siCD54 and siCD58) (n ≥ 3 independent experiments). **D:** Representative dot plots of surface expression of CD4 and CD2 on untreated and CD2 downmodulated (time point 24 h) naïve $CD4^+$ T cells (CD2mod). Data is represented as mean ± SEM. ****=p<0.0001; ***=p<0.001.

Figure S4: Cytokine secretion and expression of transcription factors in keratinocyte-T cell cocultures.

Naïve $CD4^+$ T cells were cultured for the indicated time points with untreated KCs or IFNγ-pretreated KCs loaded with (+) or without (-) SEB and then analyzed by flow cytometry. **A:** Mean values of the amounts of cytokines secreted into the supernatant

after 24 h coculture (n = 6 individual T cell donors). Professional antigen-presenting cells (pAPCs) loaded with SEB cocultures with T cells served as positive control. Cytokine secretion into the supernatant was analyzed by cytokine bead array. Not detectable cytokines are indicated by ‘n.d.’. Cytokines with a concentration below 100 pg/mL were not used for further analysis (<100 pg/mL).

B-D: Statistical evaluation of T-bet **(B)** expression, GATA3 **(C)** expression and RORγt **(D)** expression after 6 days coculture (n = 5 individual T cell donors). Intracellular transcription factor expression was analyzed after PMA/Ionomycin treatment. Data is represented as mean ± SEM. ****=p<0.0001; ***=p<0.001; *=p<0.05.

Figure S5: IL-6 production by primary human keratinocytes but not T cells and keratinocyte-mediated T-bet expression.

A: Statistical evaluation of the amount of secreted IL-6 in the supernatant of untreated KCs (white bar) or IFNγ-pretreated KCs (black bar) after 24 h (n ≥ 3 independent experiments). Cytokine secretion into the supernatant was analyzed by cytokine bead array. **B:** Intracellular IL-6 staining in naïve $CD4^+$ T cells cultured with untreated KCs (white bars) or IFNγ-pretreated KCs (black bars) (n ≥ 5 individual T cell donors). **C:** Effect of isotype control (Ctr) and blocking antibodies against CD58 and CD2 downmodulation (CD2mod) on T cells on intracellular T-bet expression in naïve $CD4^+$ T cells cultured for 6 days with untreated KCs (white bars) or IFNγ-pretreated KCs (grey bars) (n = 5 individual T cell donors). Intracellular transcription factor expression was analyzed after PMA/Ionomycin treatment. Data is represented as mean ± SEM. ***=p<0.001; *=p<0.05.

Figure S6: mRNA expression profile and STAT4 phosphorylation during keratinocyte-dependent activation of naïve CD4$^+$ T cells.

Naïve CD4$^+$ T cells were cultured for the indicated time points with untreated KCs or IFNγ-pretreated KCs loaded with SEB and then analyzed by Nanostring nCounter GEx (mRNA expression) or flow cytometry. **A:** Heat map of regulated genes in naïve CD4$^+$ T cells in a 4 h coculture (each row of either 'untreated KCs + naïve T cells' or 'IFNγ-pretreated + naïve T cells' represents data points derived from T cells from one individual donor). mRNA expression was analyzed by Nanostring nCounter human Immunology Panel.

B: Effect of isotype control antibodies (iso), blocking antibodies against CD58, or CD2 downmodulation (CD2mod) on T cells on the phosphorylation of STAT4 after 24 h coculture (n = 5 individual T cell donors). Data is represented as mean ± SEM. *=$p<0.05$.

Supplemental Experimental Procedures

Gene expression profiling

The nCounter Nanostring GX Human Immunology V2 panel was used to analyze the expression of 579 immune and inflammation associated target genes and 15 reference control genes in naïve CD4$^+$ T cells which were cultured for 4 h with either untreated or IFNγ-treated keratinocytes. Before RNA isolation, T cells were isolated using flow cytometry. Total RNA was then extracted from $1*10^6$ naïve T cells using Direct-zol RNA Miniprep Kit (Zymo Research). All RNA samples were quantified by using Qubit RNA assay kit and RNA integrity was assessed using Agilent 2100 Bioanalyzer system.

25 µg total RNA (5 µL/sample) was mixed with nCounter® reporter CodeSet (3 µL) and nCounter® capture ProbeSet (2 µL) with hybridization buffer (5 µL) for an overnight hybridization reaction at 65 °C. The reaction was cooled down to 4 °C, the

samples were purified and immobilised on a cartridge and data was assessed on the nCounter SPRINT Profiler. The exported data was analysed using NanoString nSolver 4.0.

Immunofluorescence Microscopy

Keratinocytes, either untreated or incubated with 100 ng/mL IFNγ overnight, were cultured on coverslips. These keratinocytes were loaded with SEB for 1 h at 37 °C, 5 % CO_2, washed three times with serum-free KGM-2, before naïve T cells were added in serum-free medium (XVIVO-15, Lonza). After coincubation of these cells (time points are indicated in the figures), cells were fixed with 1,5 % PFA for 20 min, permeabilized with 0,1 % saponin (in PBS + 10 % FBS) and stained with DAPI (100 ng/mL), phalloidin-AF488 (0,4 U/mL), anti-phospho-L-plastin (1 µg/mL) or anti-CD11a (LFA-1) (2 µg/mL). Anti-rb-Cy3 (1 µg/mL) was used as secondary antibody. Laser scanning confocal microscopy was performed using a Nikon A1R (40x objective; NA=1.3).

Flow cytometry

Monoclonal antibodies recognizing the following surface markers and molecules were used for flow cytometry: CD2 (RPA-2.10), CD3 (SK7), CD4 (SK3), CD8 (SK1), CD25 (2A3), CD40 (5C3), CD45RA (HI30), CD69 (L78), CD54 (HA58), CD58 (TS2/9), CD80 (L307.4), CD86 (2331), CCR3 (5E8), CCR4 (L291H4), CCR5 (REA245), CCR6 (11A9), CCR7 (150503), CCR8 (L263G8), CCR9 (L053E8), CCR10 (6588-5), CXCR3 (G025H7), IL-2 (5344.111), IL-4 (301.211), IL-6 (MQ2-13A5), IL-17A (SCPL1362), IFNγ (B27), TGFβ (TW4-9E7), HLA-DR (L243), HLA-ABC (W6/32), HLA-DQ (HLADQ1). 7AAD was used to discriminate dead cells. All antibodies were obtained from BD, eBioscience or BioLegend.

Intracellular staining for cytokines and transcription factors

Cells which were already stained for surface markers, were fixed with 1,5 % PFA for 10 min at room temperature. Intracellular staining was performed in FACS buffer (PBS, 0,5 % albumin Fraction V, 0,1 % NaN_3) containing 0,1 % saponin. Cells were washed with the same buffer and resuspended in PBS only for measurement.

For staining of transcription factors in differentiated T cells, surface markers were stained for 10 min on ice, before cells were fixed with True-Nuclear 1x Fix Concentration for 1 h at room temperature. T cells were permeabilized and stained for transcription factors (T-bet (4B10), GATA-3 (16E10A23) and RORγt (Q21-559)) in True-Nuclear 1x Perm Buffer for 30 min at room temperature.

Phospho-flow cytometry

For staining of phosphorylated proteins, surface markers were stained for 10 min on ice, before cells were fixed with Cytofix Fixation (BD Bioscience) buffer for 10 min at 37 °C, 5 % CO_2. T cells were permeabilized with Phosphoflow Perm Buffer III for 30 min on ice, before cells were stained with phospho-specific antibodies (p_{Ser727}STAT1 (A15158B), p_{Tyr705}STAT3 (13A3-1), p_{Tyr693}STAT4 (38/p-stat4), p_{Tyr705}STAT5 (47/stat5), p_{Tyr641}STAT6 (A15137E)).

Phenotyping keratinocytes

Adherent keratinocytes, either untreated or incubated with 100 ng/mL IFNγ overnight, were detached with warm trypsin/EDTA. The reaction was stopped by addition of RPMI1640 + 10 % FBS. Surface expression of HLA-DQ, HLA-DR, HLA-ABC and costimulatory receptors (CD40, CD54, CD58, CD80, CD86, and CD166) was analyzed by flow cytometry.
